# Structural Speciation of Hybrid Ti(IV)-Chrysin Systems—Biological Profiling and Antibacterial, Anti-Inflammatory, and Tissue-Specific Anticancer Activity

**DOI:** 10.3390/molecules30183667

**Published:** 2025-09-09

**Authors:** Sevasti Matsia, Georgios Lazopoulos, Antonios Hatzidimitriou, Athanasios Salifoglou

**Affiliations:** 1 Laboratory of Inorganic Chemistry and Advanced Materials, School of Chemical Engineering, Aristotle University of Thessaloniki, 54124 Thessaloniki, Greece; srmatsia@cheng.auth.gr (S.M.); glazopou@cheng.auth.gr (G.L.); 2 Laboratory of Inorganic Chemistry, School of Chemistry, Aristotle University of Thessaloniki, 54124 Thessaloniki, Greece; hatzidim@chem.auth.gr

**Keywords:** coordination chemistry, titanium, flavonoid, chrysin, inflammation, antibacterial, anticancer, in vitro investigation

## Abstract

Metal–organic compounds, and especially those containing well-known antioxidant natural flavonoids (Chrysin, Chr) and metal ions (Ti(IV)), attract keen interest for their potential biological activity nutritionally and pharmacologically. To that end, chemical reactivity profiling in binary/ternary systems was investigated synthetically, revealing unique structural correlations between mononuclear (Ti(IV)-Chr) and tetranuclear assemblies (Ti(IV)-Chr-phen). Chemical profiling involved physicochemical characterization through elemental analysis, FT-IR, UV–Visible, 1D-2D NMR, ESI-MS spectrometry, solid-state luminescence, and X-ray crystallography, with theoretical work on intra(inter)molecular interactions of 3D assemblies pursued through Hirshfeld analysis and BVS calculations. An in-depth study of their chemical reactivity shed light onto specific structural properties in the solid-state and in solution, while concurrently exemplifying quenching behavior due to their distinct flavonoid pattern. In the framework of biological activity, the materials were investigated for their antibacterial properties toward Gram(−)-*E. coli* and Gram(+)-*S. aureus*, exhibiting an enhanced effect compared to the free ligand and metal ion. Further investigation of BSA denaturation revealed strong anti-inflammatory properties compared to Chr and Diclofenac, an anti-inflammatory agent. Finally, in vitro studies using physiological and cancer cell lines, including breast (MCF10A, MCF7) and lung tissues (MRC-5, A549), formulated a structure–tissue relation reactivity profile, thus justifying their potential as future metallodrugs.

## 1. Introduction

Inflammation is an essential biological process in the human immune system [1]. It is also linked to a number of pathological aberrations, including cancer, and plays a key role in tumor development and progression. In most cases, it is characterized by physiological responses linked to immune cells, blood vessels, and molecular and cellular mediators [2,3]. As a result, chronic inflammatory diseases emerge that include metabolic syndromes, cardiovascular diseases, and cancer [4,5], among others. Remedial administration at the clinical level involves non-steroidal drugs capable of reducing or preventing inflammation [6]. Most of them, however, exhibit side-effects and undesired reactions in the gastrointestinal tract [7], cardiovascular system [8], and other organs [9,10]. Therefore, an urgent need arises to discover and develop new anti-inflammatory agents, which effectively limit inflammation, thereby opening an attractive and challenging field of biomedical research. At the same time, bacterial infections may also occur [11] during long-term inflammatory processes, thus necessitating due attention and drawing keen interest toward development of agents exhibiting concurrent antibacterial and anti-inflammatory properties.

In this context, natural products come into the forefront of research, as potential candidates of protective therapeutics, with the family of polyphenols exhibiting high biological activity against oxidative stress linked to inflammation and bacterial invasion. Representative molecules of that family are flavonoids [12,13], which are present in a wide range of plant kingdom species [14,15]. Flavones are one of the subgroups of flavonoids, exhibiting antioxidant activity, protecting cell membranes, reducing lipid levels, and inhibiting xanthan oxidase [16]. Chrysin (Chr) is a representative flavone with a broad range of biological activities [17], including anticancer, anti-inflammatory, anti-asthmatic, anti-microbial, anti-aging, anti-diabetic, anti-depressant, neuroprotective, cardioprotective, and hepatoprotective properties. Unfortunately, however, poor aqueous solubility, intensive metabolism, and low systemic absorption, lead to low bioavailability and bioactivity [18]. To confront that problem, the idea of involving metal ions and binding them to flavonoids emerges as a viable approach to achieving higher bioavailability, appropriately adjusted to elicit interactions from (sub)cellular components key to the physiology of target cells (normal or aberrant). In the process, hybrid metal–organic compounds of binary (metal ion:Chr) and/or ternary (metal ion:Chr:N,N’-chelator) nature might emerge that project modulated (bio)chemical reactivity necessary to protect healthy tissue and selectively combat disease tissue cells. In such a formulated framework, many metal ions M(II,III) have gained interest toward incorporation in novel metallodrugs as pharmaceutical agents [19], with Cu(II), Co(II), Ni(II), Mn(II), and Zn(II), having been reported to exhibit anti-inflammatory, antibacterial, antifungal, and anticancer activities [19,20]. Consequently, metal incorporation in a metal–organic drug (or organometallic) facilitates interactions with DNA and enzymes involved in processes such as gene transcription, inhibition, suppression or enhancement of metabolic and signaling pathways linked to cell survival, protection, and/or apoptotic events. The aforementioned processes and biochemical events linked to distinctly differentiated pathways thereof, constitute an integral part of our research in the lab, with select metal–organic compounds [21,22] showing merit toward applications as pharmaceutical agents, diagnostics, and therapeutic medicaments. Among the conventional metal ions used so far, titanium (Ti(IV)) has been employed as a diagnostic agent due to its low side-effects, very low allergenic potential, antibacterial properties, and low toxicity in a number of applications [23,24]. The specific metal ion (Ti(IV)) possesses attractive physical properties (charge, density, and size) that influence heavily its (bio)chemical reactivity and provide grounds for the pursuit of synthetic efforts targeting binary and ternary compounds with a biologically favorable interactive profile toward biomolecular theranostics.

Based on the above grounds, the herein presented work focuses on the development of hybrid metal–organic compounds of Ti(IV) and Chr, in binary formation or ternary assemblies involving N,N’-organic chelators, exemplifying key binding motifs capable of interacting with (sub)cellular targets, thereby affecting their state of cell physiology or disease. The synthetically isolated crystalline materials are well-characterized in the solid-state and in solution, as discrete species, with their conversion chemical reactivity supporting the broad structural speciation in the studied binary and ternary systems, further supplementing the experimentally derived profile through theoretical calculations. The collective experimental data justify further inquiry of the emerging materials as potential antibacterial (Gram(−) (*E. coli*) and Gram(+) (*S. aureus*) bacteria) and anti-inflammatory agents (BSA denaturation assay). In that respect, a well-formulated (bio)chemical profile is generated for well-defined hybrid complex species of Ti(IV) and flavonoids that merit further investigation as competent and effective metallopharmaceuticals.

## 2. Results

### 2.1. Synthesis of Binary and Ternary Materials

**Scheme 1:**
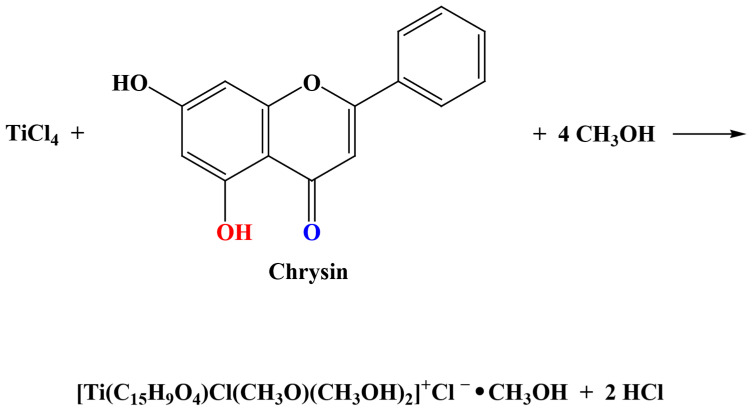
Reactivity of binary Ti(IV)-Chr system, leading to compound **1**. Potential metal binding sites are noted through different color terminal atoms in the flavonoid structure.

The synthesis of the binary compound [Ti(C_15_H_9_O_4_)(CH_3_O)Cl(CH_3_OH)_2_]Cl • CH_3_OH (**1**) and the ternary compound [Ti_4_O_4_(C_15_H_9_O_4_)_4_(C_12_H_8_N_2_)_4_]Cl_4_•2CH_3_OH• 5(CH_3_)_2_CHOH (**2**) was pursued through facile chemical reactivity work in alcoholic media. Specifically, compound **1** (**Ti(IV)-Chr**) was synthesized from the binary system of Ti(IV) and Chr in alcoholic media (methanol) with a molecular stoichiometry of titanium:chrysin 1:1. The overall reaction leading to **1** is shown in Scheme 1.

**Scheme 2:**
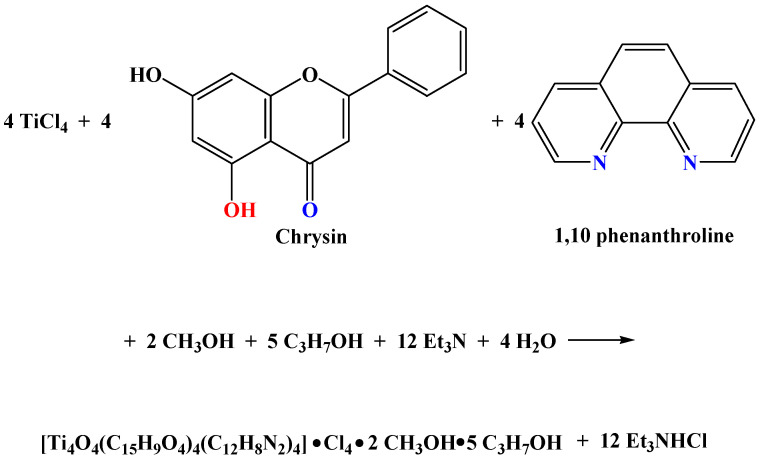
Reactivity of ternary Ti(IV)-Chr-phen system, linked to the synthesis of compound **2.** Potential metal binding sites are noted through different color terminal atoms in the flavonoid structure and the aromatic chelator.

Compound **2 (Ti(IV)-Chr-phen)** emerged from a ternary system of Ti(IV), Chr, and the N,N’-aromatic chelator (1,10-phenanthroline, phen) with a molar ratio of titanium:chrysin:phen 1:1:1. In this case, the reaction was pursued in a basic environment, using triethylamine as a Lewis base. The reaction linked to the synthesis of **2** is depicted in Scheme 2.

### 2.2. Transformation of ***1*** to ***2***

**Scheme 3:**
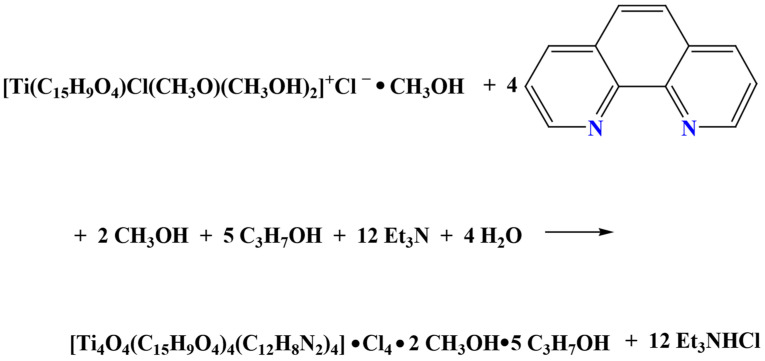
Transformation of Ti(IV)-Chr (**1**) to Ti(IV)-Chr-phen (**2**). Potential metal binding sites are noted through different color terminal atoms in the aromatic chelator structure.

Driven by the fact that the ternary system of compound **2** is a tetranuclear assembly, comprised of the binary system of Ti(IV):Chr, further enriched by the ancillary N,N’-aromatic chelator phen, chemical reactivity was considered that could link the two species **1** and **2** upon introduction of the aromatic chelator phen to **1**. To that end, efforts were made to associate the tetranuclear assembly in **2** with the architecture in **1**, with the latter species serving as starting material. Consequently, **1** was employed in a reaction introducing phen in a basic environment, ultimately leading to **2** as shown in Scheme 3.

The derived binary (**1**) and ternary (**2**) materials were retrieved easily from the reaction mixtures in pure crystalline form. Elemental analysis of the isolated crystalline products projected the molecular formulation [Ti(C_15_H_9_O_4_)(CH_3_O)Cl(CH_3_OH)_2_]Cl • CH_3_OH and [Ti_4_O_4_(C_15_H_9_O_4_)_4_(C_12_H_8_N_2_)_4_]Cl_4_•2CH_3_OH• 5(CH_3_)_2_CHOH, respectively. Further spectroscopic evaluation of the crystalline products by FT-IR confirmed the presence of ligands bound to Ti(IV), thus being in line with the proposed formulation. Finally, X-ray crystallography confirmed the analytical and spectroscopic results by divulging the molecular formulation of the crystalline products in all cases. Compounds **1** and **2** are sparingly soluble in water, and are stable in the crystalline form in the air at room temperature for long periods of time.

### 2.3. Description of X-Ray Crystallographic Structures

**Figure 1:**
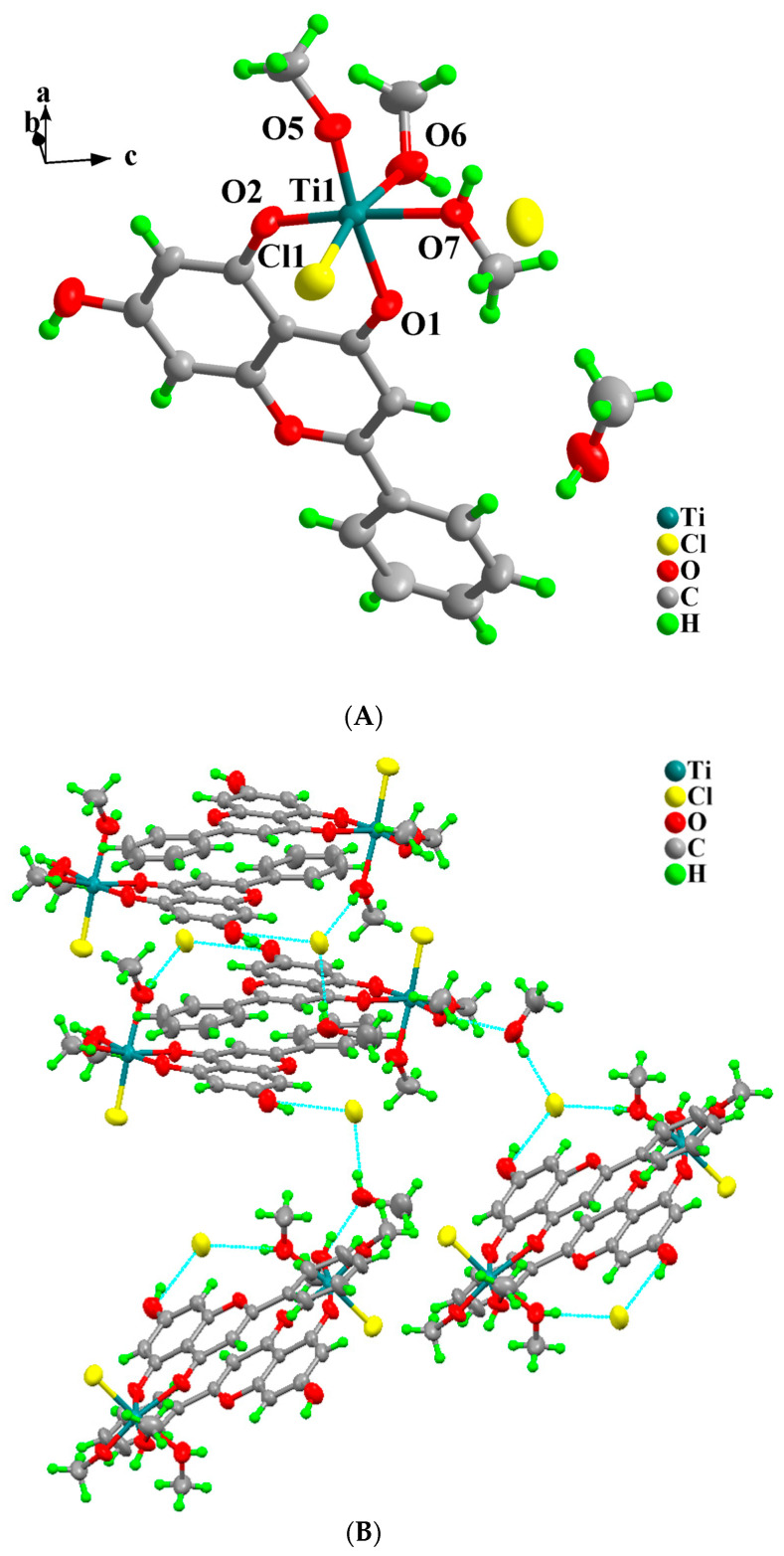
(**A**) Molecular structure of the complex assembly in the asymmetric unit of **1** in ORTEP; (**B**) Hydrogen-bonding interactions in the structure of **1** (ORTEP), displayed as blue dotted lines.

Compound **1** crystallizes in the monoclinic crystal system and space group *P*2_1_/*n*, with Z = 4. The asymmetric unit of complex **1** emerges from a mononuclear singly cationic Ti(IV)-Chr assembly, one chloride counter anion, and one solvate methanol molecule. The metal assembly cation is formulated as [Ti(C_15_H_9_O_4_)(CH_3_O)Cl(CH_3_OH)_2_]^+^, containing the central metal ion Ti(IV), one singly deprotonated Chr anion, one methoxido anionic ligand, one coordinated chloride anion, and two bound methanol molecules. The Chr anion acts as a bidentate chelate ligand, coordinated through the deprotonated phenolic group and the carbonyl oxygen atoms. The process leads to a six-coordinate Ti(IV) metal center with a distorted octahedral coordination sphere. The most axial vector is drawn through O(2)-Ti-O(7), with the remaining three oxygen atoms together with the chloride ion forming the equatorial plane. Oxygen atom bond lengths to Ti(IV) vary from 1.7397(15) to 2.1005(17) Å, with the shortest distance involving the methoxido oxygen O(5) and the longer ones being the bond distances corresponding to the Ti(IV)-bound methanol oxygen atoms. Figure 1A shows the structure of complex cationic assembly in **1**. Table 1 contains the experimental parameters linked to data collection and structure refinement. Selected interatomic distances and bond angles are provided in Table 2.

Hydrogen-bonding interactions bestow additional stability upon the assembled lattice, in view of the fact that the chloride counter ion interacts in a Y fashion with three hydrogen atoms originating from the protonated phenolic group and the hydroxy group of one ligated methanol molecule from a neighboring complex as well as the solvated methanol molecule. The latter molecule is also interacting with the hydrogen from the second bound methanol molecule, finally giving rise to an extended 3D lattice, as shown in Figure 1B. Metal center oxidation state assignment was supported through BVS calculations, revealing a value of 3.901 for Ti(IV).

**Figure 2:**
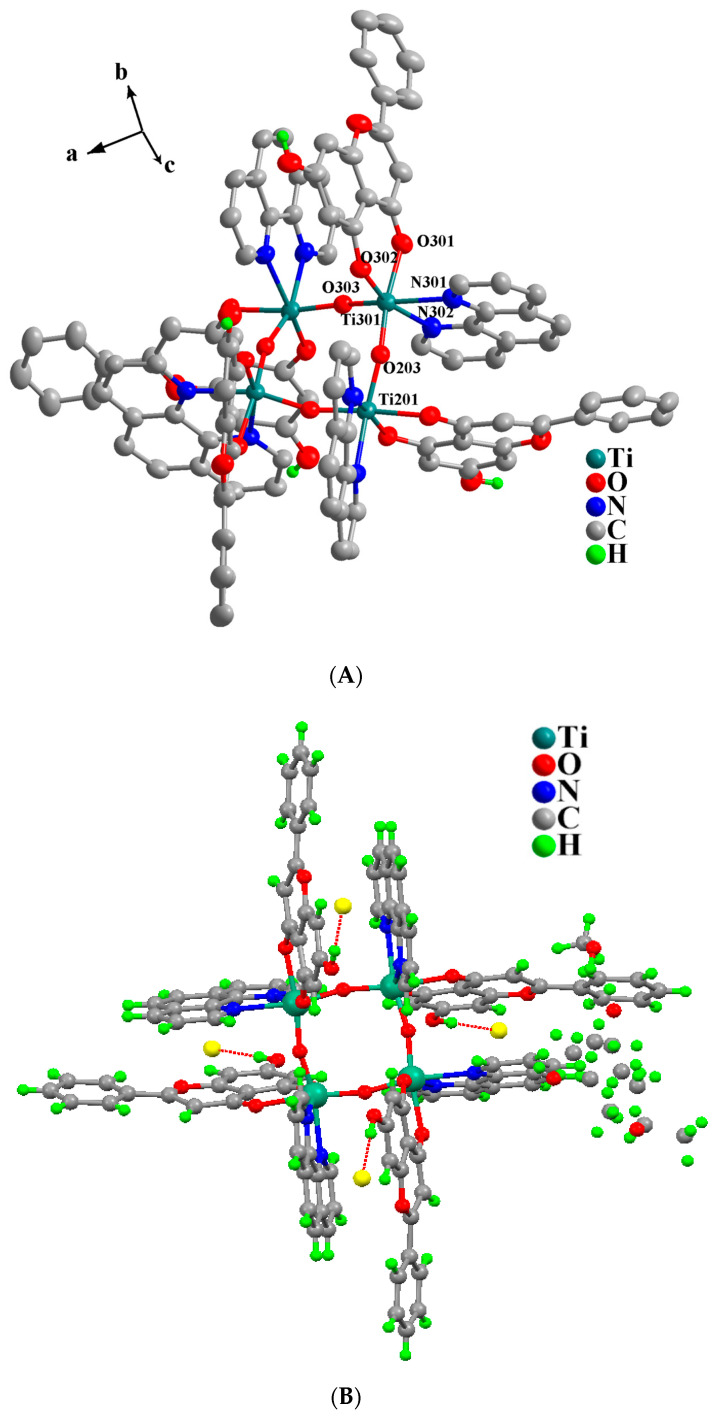
(**A**) Molecular structure of **2** (ORTEP). Aromatic hydrogen atoms, disordered atoms, and solvate molecules are omitted for clarity; (**B**) Hydrogen-bonding interactions in **2** (red dotted lines in ORTEP assembly).

Compound **2** (Figure 2A) crystallizes in the tetragonal crystal system and space group *P*4_2_/*n*, with Z = 2. The unit cell contains two tetranuclear Ti(IV) assemblies and eight chloride counter anions, and a total of ten solvated isopropanol and four solvated methanol molecules. All of the solvate molecules were found disordered. The main tetranuclear Ti(IV) complex assembly is symmetry-generated, since the asymmetric unit contains only one Ti(IV). In the asymmetric unit, in addition to the central metal ion, one Chr deprotonated anion, one phen ligand, and two bridging oxygen atoms were found. These bridging oxygen atoms (O(3) and the symmetry-generated one) connect in pairs all four Ti(IV) cations, thus giving rise to the final tetranuclear cationic assembly bearing a total charge of 4+. All bridging oxygen atoms are placed in cis positions around the metal ions. Both phen and Chr moieties are acting as bidentate chelate ligands. Finally, each Ti(IV) center adopts a coordination number of six, thus generating a distorted octahedral coordination sphere. Figure 2A shows the molecular structure of **2**, and Table 2 contains selected bond distances and angles. Hydrogen-bonding interactions, keeping in close proximity the chloride counter anions and the protonated phenolic parts of the four Chr ligands as well as the solvate alcoholic molecules, bestow stability upon the system and form a 3D crystal lattice, as shown in Figure 2B. BVS calculations for the metal center revealed a value of 3.851 for Ti(IV).

### 2.4. Hirshfeld Surface Analysis

**Figure 3:**
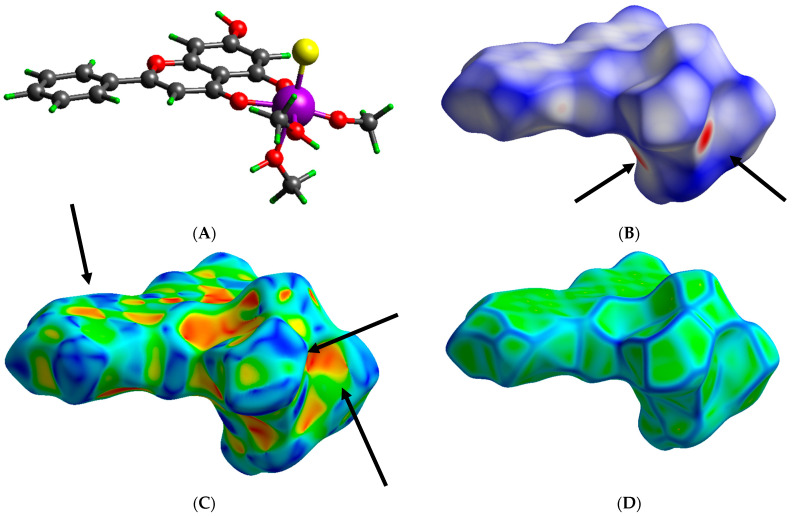
(**A**). Crystal Explorer of **1**; mapping through (**B**) d_norm_; (**C**). Shape index; and (**D**). Curvedness. Explanatory information on arrows is provided in the text.

In order to investigate and further evaluate the solid-state structural properties emanating from the aforementioned structures and 3D assemblies, Hirshfeld surface analysis has been pursued for the delineation of intermolecular and intramolecular interactions. Interactions were visualized through 3D mapping, using d_norm_, Shape index, and Curvedness surface analysis, and were further quantified using 2D Fingerprint plots for their proportional determination though deconvolution of Full plot. Specifically, as shown in Figure 3A–D, for the binary system Ti(IV)-Chr (**1**), in the case of d_norm_ mapping (Figure 3B), there are overall two red spots (black arrows) pointing toward bound methanol moieties, showing close O–H···O hydrogen bonds with methanol and O–H···Cl contacts with the chloride ion in the lattice [25]. The remainder of the molecule shows contacts close to van der Waals radii (white areas) or longer ones (blue areas) [26] all around the Chr ligand. In Shape index surface, a “bow-tie’’ pattern (black arrows) of touching blue–red triangles (Figure 3C) is shown, indicating the existence of weak C–H···π interactions between adjacent Chr ligands. Red depressions of concave curvature (black arrows) show close contact interactions corresponding to hydroxyl groups, while blue regions of the opposite curvature (convex) (black arrows) show close contact regions between the moieties of the same molecule and unique close interactions corresponding to C–H donor-like contacts of methyl groups. Blue concave regions over Chr rings and methanolic moieties attest to the presence of donor-like C–H interactions with lattice methanol and/or Chr molecules. The overall interactions, as also shown in Figure 3D through Curvedness mapping, are shown to be more active on the metal-bound methanol, methoxido groups, and the chloride ion. Sharp curvature areas, separated by blue edges on that site, indicate interactions between neighboring molecules, with the other site (Chr site) exhibiting more flat regions with low intensity of blue edges, thus indicating lower activity of interactions.

**Figure 4:**
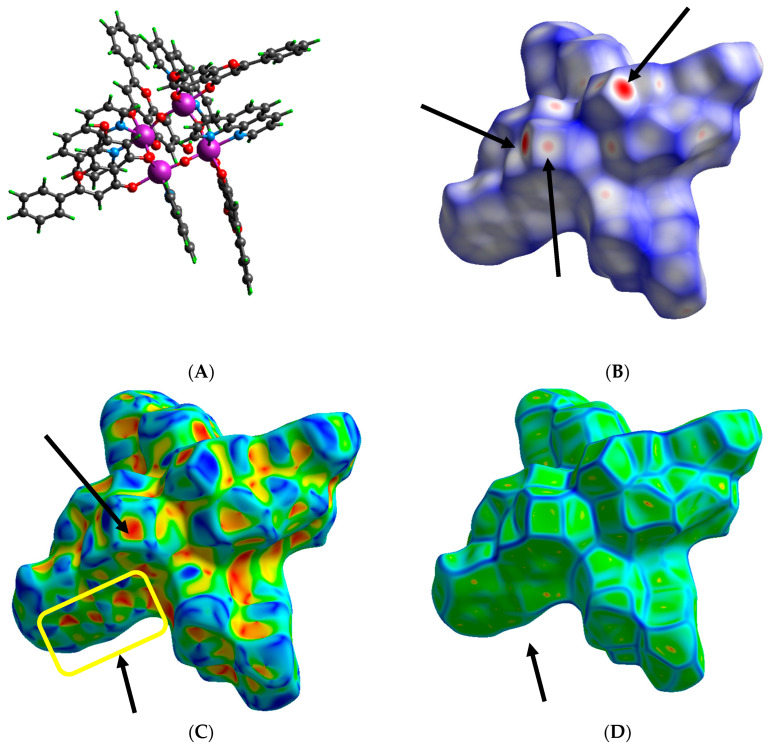
(**A**). Crystal Explorer of **2**; mapping through (**B**) d_norm_; (**C**). Shape index; and (**D**). Curvedness. Explanatory information on arrows is provided in the text.

In the case of the tetranuclear assembly of the ternary Ti(IV)-Chr-phen (Figure 4A) compound, mapping over the three different types of surfaces (Figure 4B–D) reveals that Chr is the most important molecular competitor on close contact interactions.

**Figure 5:**
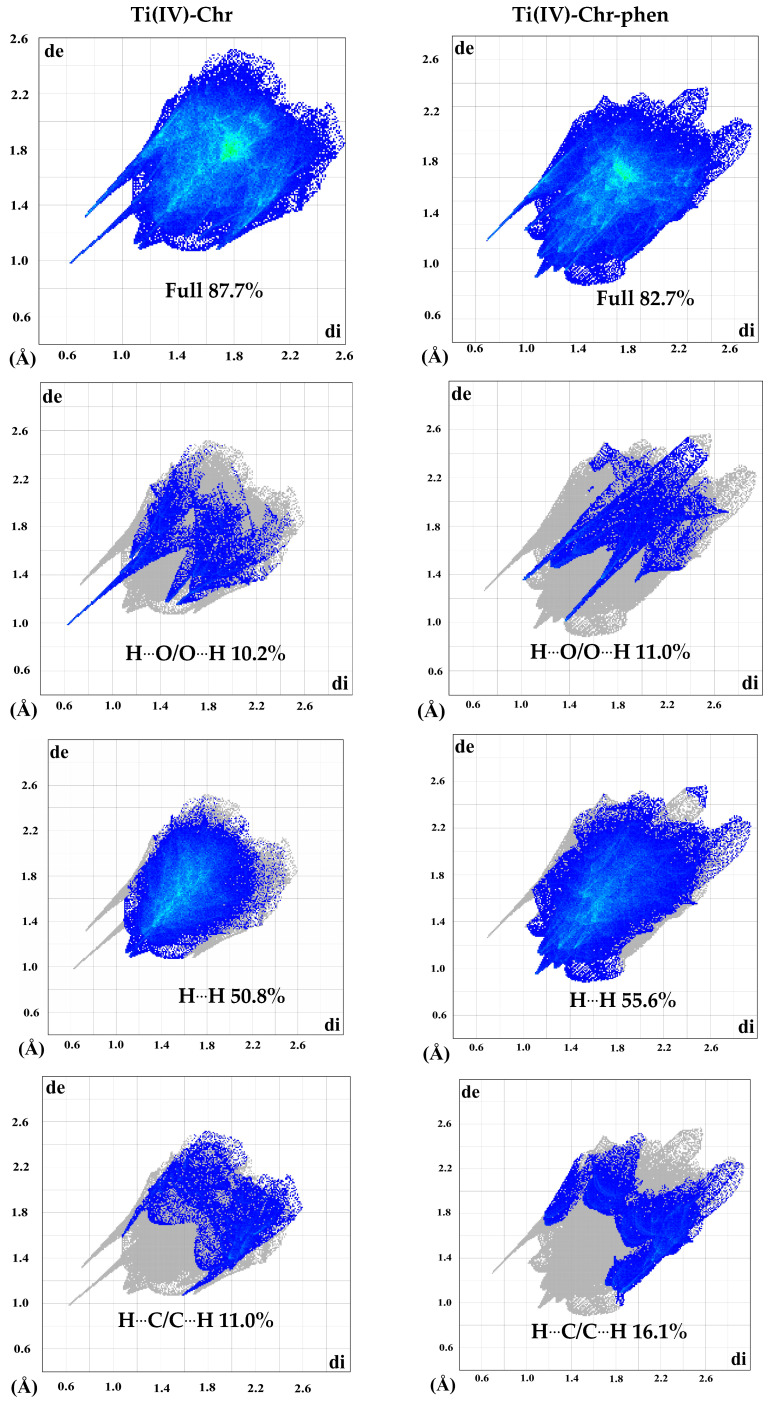
2D Fingerprint plots and their deconvolutions for **1** and **2.** Blue and gray colorations shown in the plots are explained in the text.

Specifically, the phenolic group of the metal-bound Chr, as shown in d_norm_ with deep red spots (black arrows) (Figure 4B), confirms the close contact of O–H···Cl interactions with the chloride ion in the lattice. Lower intensity red circles (black arrows) indicate weaker or stronger contacts of C–H interactions, also corresponding mostly to C–H groups of the bound Chr ligand. Large white areas over the Chr ring reflect contacts close to van der Waals radii, whereas the rest of the molecule is depicted with blue regions, showing that the phen-bound molecule is involved in longer contacts. Shape index mapping also verifies the aforementioned interactions by concave red regions (black arrows) on phenolic groups in Chr, showing donor-like O–H···Cl interactions and blue–red opposite triangles of “bow-tie’’ pattern (yellow rectangle) on the pyran and phenyl rings of Chr, thus verifying planar stacking arrangements. The third ring of Chr, also containing the phenyl group and the phen-bound moiety, shows a sharper curvature in both Shape index and Curvedness (blue edges area) surfaces, thus verifying their participation in C–H contacts with lattice methanol groups. Yellow and red spots, in the case of Curvedness on flat regions (black arrow) and in the sharp curvatures, also verify the existence of H···H bonding interactions. In addition, internal vs. external interactions have been quantified through 2D Fingerprint plots and deconvoluted for further calculation of distinct interactions for **1** and **2**, as shown in Figure 5. The Hirshfeld surfaces displayed quite clearly the fact that O–H···O, H···H, and H–C···H interactions are the most abundant ones in both assemblies. Specifically, O–H···O interactions stand at 10.2% in Ti(IV)-Chr and 11% in Ti(IV)-Chr-phen, reflected as sharp spikes (blue area) characteristic of donor–acceptor interactions. The remaining area, not contributing to the specific interactions, is shown as densely interspersed gray points.

The most dominant interactions correspond to H···H contacts, shown as diffuse areas of blue points in the deconvoluted plot, comprising 50.8% of the binary and 55.6% of the ternary system. Moreover, a pair of wings (blue area) point out the contribution of C–H···C interactions reaching 11.0% (Ti(IV)-Chr) and 16.1% (Ti(IV)-Chr-phen). In the case of the Ti(IV)-Chr assembly, there is a characteristic pair of sharp spikes (blue area) attributed to H···Cl interactions with the bound and/or lattice chloride ion at a level of 15.7%, as shown in Figure S1.

### 2.5. FT-IR Spectroscopy

Solid-state FT-IR spectra (4000 to 400 cm^−1^) were recorded on KBr pellets for compounds **1**–**2**. FT-IR patterns of studied compounds have been compared with those of the free ligands (Chr and phen). The spectra show vibrations corresponding to Chr and phen in a broad spectral range from ~1600 to ~450 cm^−1^ as well as at ~3500 cm^−1^. Specifically, there is a strong shift in the flavonoid carbonyl (C=O) group from 1653 cm^−1^ (high intensity peak in the free ligand) to 1634 cm^−1^ for **1** and 1625 cm^−1^ for **2**. Vibrational modes attributed to ν(C–O–C) and ν(C=C) at 1347 cm^−1^ for free Chr exhibit a slight shift for both metal–organic compounds (1360 cm^−1^ for **1** and 1352 cm^−1^ for **2**), thus verifying that the oxygen atom of the heterocyclic pyran ring is not bound to the metal center [27]. There is also a differentiation in the patterns related to symmetric and antisymmetric O-H stretching modes, indicating deprotonation of the flavonoid and its concomitant coordination to the titanium metal center [28,29]. Specifically, what is shown is a shifted broad band at 3531 cm^−1^ and 3389 cm^−1^ in the case of **1**, and 3387 cm^−1^ in the case of **2**, respectively, compared to that of free Chr shown at 3431 cm^−1^. In the case of the phen ligand, sharp peaks can be observed at frequencies 1429 and 1511 cm^−1^, attributed to the presence of ν(C=C) and ν(C=N) bonds, respectively. In the case of the ternary Ti(IV)-Chr-phen system, phen-related vibrations have been shown at 1420 cm^−1^ and 1525 cm^−1^. Low intensity bands, observed in the range 400–600 cm^−1^, are attributed to ν(Ti(IV)–N) and ν(Ti(IV)–O), thus confirming metal–ligand bond formation [30,31]. All of the aforementioned spectral features are in agreement with the structures of compounds **1** and **2**, revealed by X-ray crystallography.

### 2.6. UV–Visible Spectroscopy

**Figure 6:**
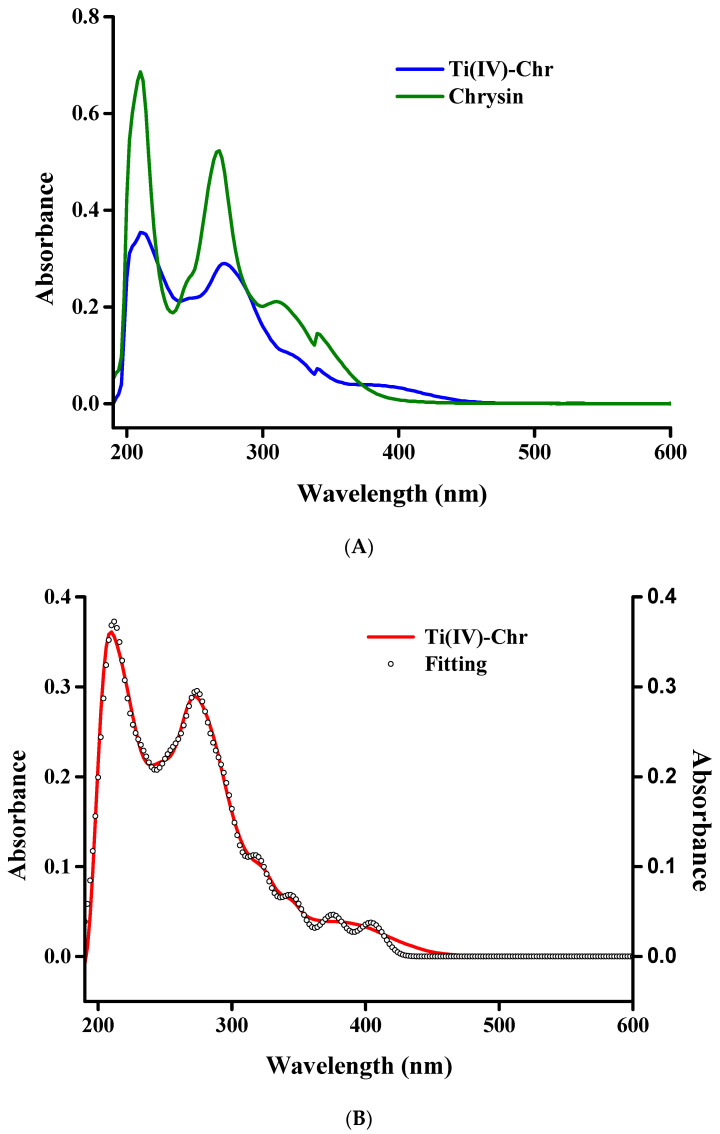
(**A**). UV–Visible spectra of **1** compared to Chr in methanol at 10^−5^ M; (**B**). Fitting spectra of **1**.

UV–Visible electronic spectra were recorded in methanol, in the wavelength range 190–600 nm. The arising absorption features of the studied compounds **1**–**2** were compared with free Chr and phen ligands (Figure 6 and Figure S2). Recorded spectra were further fitted using the Savitzky–Golay algorithm, thus verifying that the absorption peaks to be analyzed are detected on the pattern of the spectra, and there are no artifacts. Characteristic absorption peaks of Chr attributed to π-π* transitions of the benzoyl and cinnamoyl systems are shown at 210 nm, 267 nm, and 310 nm. The aforementioned peaks are slightly shifted to 210 nm (ε 33,143 M^−1^•cm^−1^), 269 nm (ε 269,971 M^−1^•cm^−1^), and 321 nm (shoulder). A new broad absorption was shown at 392 nm (ε 5976 M^−1^•cm^−1^), attributed to Ligand to Metal Charge Transfer (LMCT) transitions, owing to binding of the metal center to the carbonyl group of the flavonoid. In the case of **2**, the following peaks at 210 nm (ε 200,729 M^−1^•cm^−1^), 269 nm (ε 216,500 M^−1^•cm^−1^), and 319 nm (shoulder) emerged shifted and with higher intensity. A Gaussian-like absorption also appeared at 395 nm (ε 24,513 M^−1^•cm^−1^). The N,N’-organic chelator (phen) absorption pattern was also recorded in methanol, thus providing two high intensity peaks at 212 and 269 nm as well as a shoulder-like absorption at 318 nm, corresponding to n → π* and π → π* transitions. The overall pattern of both compounds suggests that the Chr and phen ligands are bound to Ti(IV), as a result of which a slight shift in the absorption occurs that is related to π-π* transitions and the appearance of new broad bands corresponding to LMCT effects and n → π* transitions. All of the aforementioned characteristic absorptions are in line with other flavonoid-based compound spectral features and patterns [28,32,33].

### 2.7. ESI-MS Analysis

Positive mode ESI-MS spectra of compounds **1** and **2** were recorded in methanolic solutions at a concentration 10^−4^ M. The spectral patterns are shown in Figure S3A for **1** and Figure S3B for **2**. Specifically, the spectrum of **1** exhibits the following species: M1_1 = [M_1_-Cl-CH_3_OH-H] = [C_17_H_15_TiO_6_]^+^, *m*/*z* = 361.0369–365.0311 (z = 1); M1_2 = [M1 + 4CH_3_O + Cl-5H] = [C_22_H_27_TiCl_2_O_11_]^9−^, *m*/*z* = 580.8574–587.0616 (z = 1). The spectrum of **2** reveals more species, as follows: M2_1 = [M2/4-phen-O + 2CH_3_O] = [C_17_H_15_TiO_6_]^+^; *m*/*z* = 361.0374–365.0288 (z = 1); M2_2 = [M2/4] = [C_27_H_17_TiN_2_O_5_]^+^, *m*/*z* = 495.0640–499.0518 (z = 1); M2_3 = [M2/2-phen-H] = [C_42_H_25_Ti_2_N_2_O_10_]^+^, *m*/*z* = 812.0477–814.0453 (z = 1); M2_4 = [M2/2-H] = [C_54_H_33_Ti_2_N_4_O_10_]^+^, *m*/*z* = 992.1186–995.1147 (z = 1); M2_5 = [3M2/4-O-3phen + 3CH_3_OH-H] = [C_48_H_38_Ti_3_O_17_]^4+^, *m*/*z* = 1029.1116–1032.1080 (z = 1); M2_6 = [M2-2phen-2Chr+7H] = [C_54_H_41_Ti_4_N_4_O_12_]^13+^, *m*/*z* = 1128.0333–1132.0308 (z = 1). Absolute error values (*m*/*z* differences) have been calculated for all of the aforementioned species and are shown in Table S1 [34].

### 2.8. NMR Spectroscopy

#### 2.8.1. 1D ^1^H-, ^13^C-NMR

**Figure 7:**
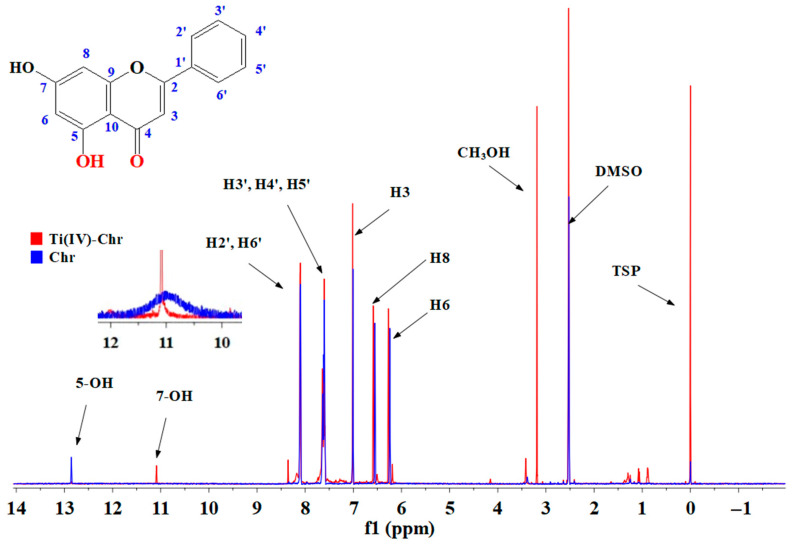
^1^H PRESAT NMR spectra of **1** in DMSO-d^6^.

**Figure 8:**
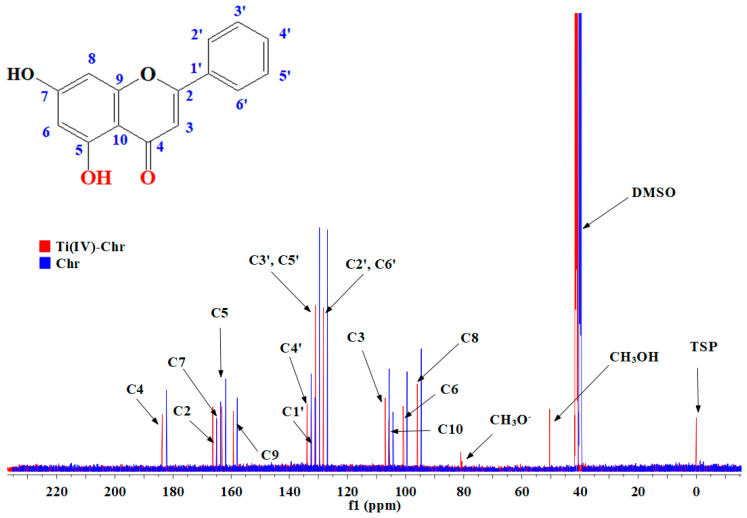
^13^C NMR spectra of **1** in DMSO-d^6^. All carbon atoms bear their distinct signature, emanating from their position in the structure of the molecule depicted in the spectrum.

1D NMR spectra of compounds **1**–**2** (Figure 7, Figure 8 and Figure S4A,B) were recorded in DMSO-d^6^, and characteristic signal and spectral patterns were further compared to that of free Chr and phen ligands. Specifically, the ^1^H-NMR (PRESAT) patterns of **1** and **2** show no significant shifts (0.01–0.02 ppm) compared to that of free Chr in the range 8.11–6.27 ppm, attributed to the aromatic ligand of Chr. In the free Chr ligand, the spectrum shows a single peak at 12.85 ppm, corresponding to the OH group bound to C(5). The peak at 10.95 ppm, shown as a broad band, is attributed to the OH group bound to C(7). In both compound spectra, there is no peak related to the OH moiety at C(5), and a single peak appears to have shifted significantly at 11.09 ppm for **1** and 11.12 ppm for **2**, thus verifying the deprotonation of the phenyl-C(5) group and further coordination to the central metal ion [35,36,37,38]. Furthermore, in the case of **1**, there is a strong single peak at 3.19 ppm, linked to the bound methanolic moiety. Comparison of the compound **2** pattern with that of free phen shows a total downfield shift for multiple peaks. All of the aforementioned shifts in the proton spectra are shown in Table S2 in comparison to the free ligands.

The ^13^C-NMR spectra of both compounds show significant shifts associated with the flavonoid and phen ligands. Every carbon peak was noticeably shifted downfield for **1** compared to the free ligands, and variably downfield and upfield for **2**.

All peaks are reported in Table S3. Significant de-shielding was shown for C(5) (by 1.36 ppm for **1** and 1.11 for **2**) and C(4) (by 1.41 ppm for **1** and 1.29 for **2**). Further, significant shifts were exhibited for C(2) (by 1.40 ppm for **1** and 2.90 ppm for **2**), C(7) (by 1.32 ppm for **1** and 3.48 ppm for **2**), C(8) (by 1.40 ppm for **1** and 1.49 ppm for **2**), and C(9) (by 1.44 ppm for **1** and 1.00 ppm for **2**). Benzoyl ring carbon shifts were shown to be shifted downfield (by ~1.40 ppm) for **1** and (by ~1.20 ppm) for **2.** All of the aforementioned features are consistent with participation of the C(5)–O moiety in metal ion chelation. There are also downfield and upfield shifts related to C(11)-C(22) (from 150.86 to 127.90 ppm) of the bound phen molecule. In the spectra of **1**, there are also peaks (singlet) linked to the bound methanol and methoxido moieties at 50.24 and 81.00 ppm, respectively. Proton and carbon resonances of **1** and **2** shifted considerably downfield as well as upfield, thus specifically attesting to that aromatic ring of the Chr ligand being coordinated to Ti(IV). In the case of the ternary system in **2**, phen-based resonances have been shown to have shifted compared to that of free phen ligand [39,40]. Specific assignments related to the correlation of proton and carbon nuclei have been determined through 2D NMR.

#### 2.8.2. 2D gCOSY and gHSQC NMR

**Figure 9:**
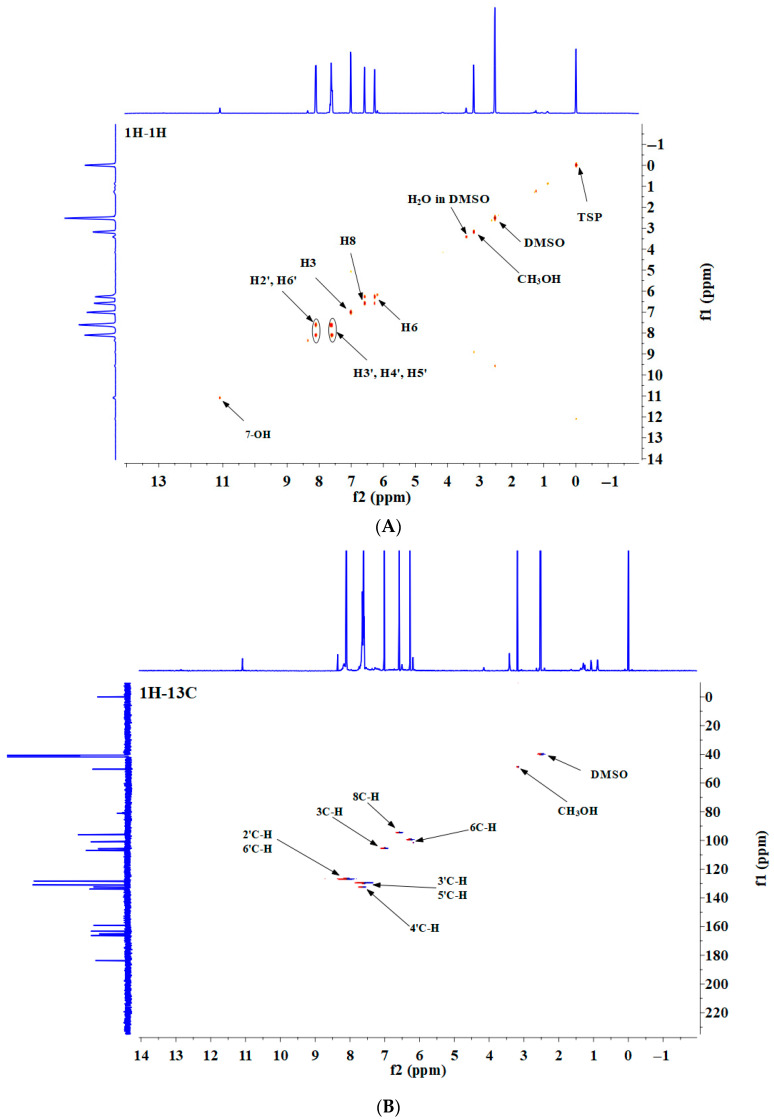
(**A**) ^1^H-^1^H correlation through gCOSY NMR of **1**; (**B**) ^1^H-^13^C correlation through gHSQC NMR of **1**.

2D NMR spectra were recorded in DMSO-d^6^ and the appropriate correlations are shown in Figure 9A,B and Figure S5A,B. Homonuclear and heteronuclear correlations were studied for further identification of the new Ti(IV) species. To that end, ^1^H-^1^H and ^1^H-^13^C correlation spectra were collected through pulsed field gradients for enhanced resolution and low signal-t-noise ratio.

Gradient Correlation Spectroscopy (gCOSY) experiments (Figure 9A and Figure S5A) showed no cross peak downfield, thus verifying that there is deprotonation of the OH group bound to the C(5) atom. Further, in the range 8.20–7.00 ppm, there are cross peaks for all H-H assignments, confirming the coupling patterns of the B ring in the Chr moiety bound to Ti(IV). That way, it is verified that the flavonoid ligand maintains the overall structure in the metal–organic complex. Hydrogen interactions, revealed through the presence of the methanolic solvent, are also ostensible. The Gradient Heteronuclear Single Quantum Coherence (gHSQC) spectra of **1**–**2** show independent peaks, which correlate C(3), C(6), and C(8) carbons with the appropriate hydrogen atoms, thus showing that the assignments are slightly shifted and yet remain correlated. Further verification of specific sites of the Chr molecule bound to Ti(IV) was also revealed.

### 2.9. Luminescence Studies

**Figure 10:**
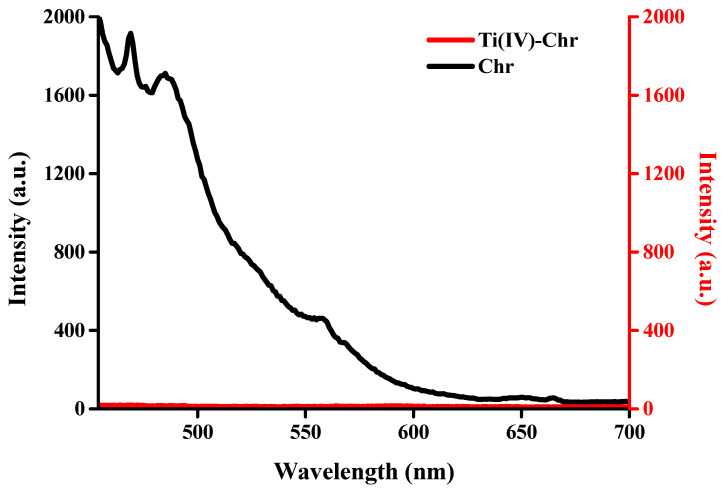
Solid-state normalized luminescence spectra of **1** compared to Chr.

Solid-state luminescence experiments on compounds **1**–**2** were carried out at room temperature. The excitation spectrum of **1** (Figure 10) shows lower luminescence activity (quenching) compared to that of free Chr. Specifically, quenching was observed at λ_em_ 469 nm (λ_ex_ 375 nm). The same quenching effect was observed in the luminescence spectrum of **2** (Figure S6A,B) compared to free Chr at λ_em_ 469 nm (λ_ex_ 363 nm) and the phen organic chelator at λ_em_ 418 nm (λ_ex_ 356 nm). This specific effect is in accordance with all previously studied metal–organic flavonoid-based systems [21,22,28].

### 2.10. Antibacterial Properties

Investigation of the antibacterial potential of compounds **1**–**2** was pursued through the employment of the disk diffusion method in Gram(−) (*E. coli*) and Gram(+) (*S. aureus*) bacterial strains. Zone Of Inhibition (ZOI) values were determined in a concentration-dependent study and Minimal Inhibitory Concentration (MIC) values, expressed as mass in mg/cm^2^, were derived and are shown in Table 3. The antibacterial efficacy of both compounds was compared to that of free flavonoid, Chr, and the aromatic chelator activity, with respect to concentration adjusted, on a molar scale, to that of the investigated metal–organic compound. The flavonoid, Chr, shows no inhibition of bacterial growth up to 0.25 mg (0.88 mg/cm^2^) for *E. coli* and 5.0 mg (17 mg/cm^2^) for *S. aureus*. In the case of the aromatic chelator phen, the determined ZOI values are 20.5 ± 0.3 mm for *E. coli* and 30.2 ± 0.2 mm for *S. aureus*. Titanium oxide shows a ZOI value of 29.7 ± 0.2 mm (0.080 mg (0.28 mg/cm^2^) in *E. coli* and no inhibition in *S. aureus* up to a concentration of 1.5 mg (5.3 mg/cm^2^)). The binary, Ti(IV)-Chr, metal–organic compound **1** exhibited an inhibition value of 31.5 ± 0.1 mm at 0.50 mg (1.8 mg/cm^2^) for *E. coli* and 13.5 ± 0.2 mm at 10 mg (35 mg/cm^2^) for *S. aureus*. The ternary system of Ti(IV)-Chr-phen in **2** shows ZOI values of 27.5 ± 0.1 mm at 0.080 mg (0.28 mg/cm^2^) for *E. coli* and 15.3 ± 0.1 mm at 10 mg (35 mg/cm^2^) for *S. aureus*, thus providing a structure-specific profile of antibacterial activity for both materials against Gram(−) bacteria.

### 2.11. Anti-Inflammatory Activity

**Figure 11:**
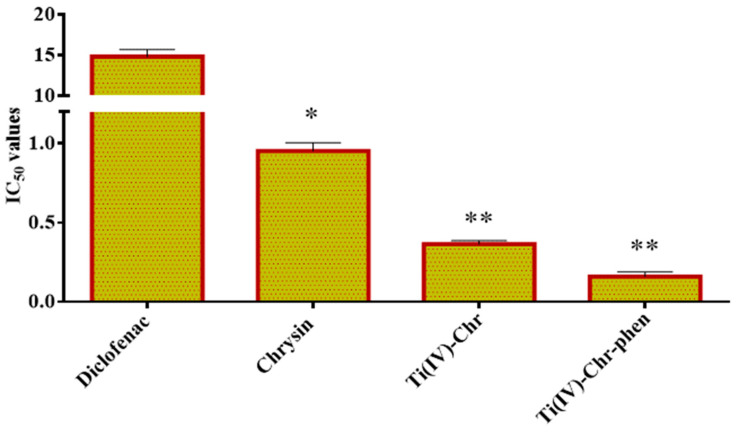
Anti-inflammatory activity of **1** (**Ti(IV)-Chr**) and **2** (**Ti(IV)-Chr-phen**) in comparison to a reference drug and neat flavonoid. Significance levels were assessed as follows: * *p* < 0.05 (significant), ** *p* < 0.01 (highly significant), *** *p* < 0.001 (extremely significant), and **** *p* ≤ 0.0001 (extremely significant).

The anti-inflammatory activity was examined through the Bovine Serum Albumin (BSA) denaturation assay. Specifically, compounds **1**–**2** were studied for their ability to counteract the heat-induced denaturation of BSA and were further expressed as IC_50_ values (μg/mL). The activity of Chr, as a control compound, was examined in the range 0.001 μg/mL–20 μg/mL. IC_50_ values were compared with a known reference anti-inflammatory drug (Diclofenac sodium salt) and to that of the free Chr ligand. Compound **1** shows anti-inflammatory activity at 0.364 ± 0.031 μg/mL and compound **2** exhibits a lower value at 0.160 ± 0.039 μg/mL. Both compounds, as shown in Figure 11, show highly significant anti-inflammatory activity compared to that of free Chr. Both compounds (**1**, **2**) and free Chrysin reveal a significant difference compared to diclofenac (IC_50_ = 14.8 ± 1.3 μg/mL), revealing *p* values at 0.0095, 0.0089, and 0.0101, respectively.

### 2.12. Cytotoxicity Results

#### 2.12.1. Cell Viability

**Figure 12:**
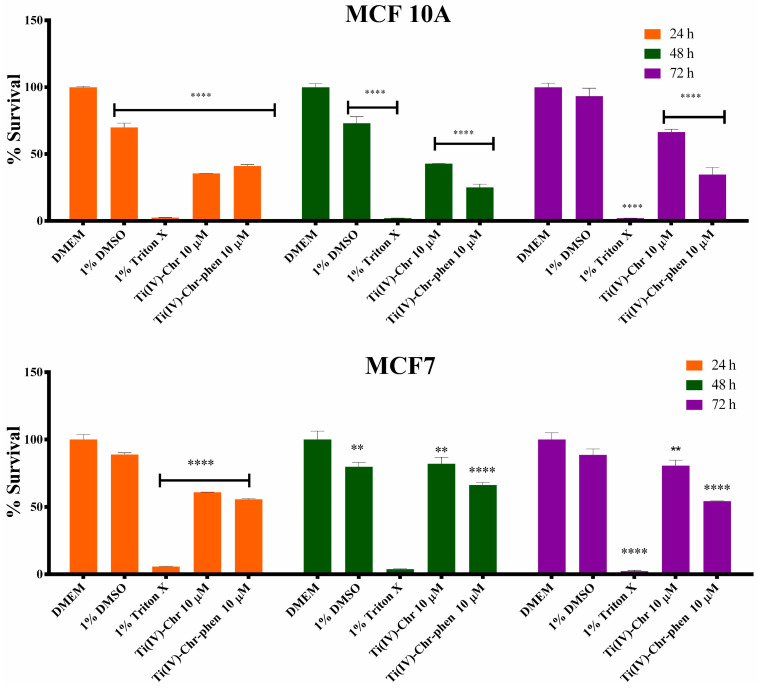
Viability assessment for compounds **1** (**Ti(IV)-Chr**) and **2** (**Ti(IV)-Chr-phen**), in physiological (MCF10A) and cancer (MCF7) human breast tissue cell lines. Significance levels were assessed as follows: * *p* < 0.05 (significant), ** *p* < 0.01 (highly significant), *** *p* < 0.001 (extremely significant), and **** *p* ≤ 0.0001 (extremely significant).

**Figure 13:**
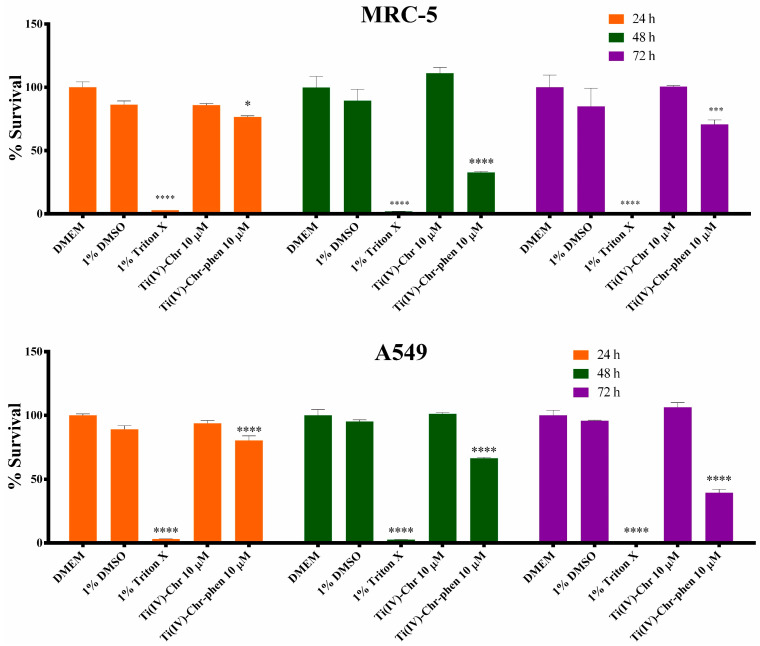
Viability assessment for compounds **1** (**Ti(IV)-Chr**) and **2** (**Ti(IV)-Chr-phen**), in physiological (MRC-5) and cancer (A549) human lung tissue cell lines. Significance levels were assessed as follows: * *p* < 0.05 (significant), ** *p* < 0.01 (highly significant), *** *p* < 0.001 (extremely significant), and **** *p* ≤ 0.0001 (extremely significant).

The new metal–organic compounds were investigated for their (a) toxicity profile (Figure 12 and Figure 13), through study of the viability of cells treated with compounds **1**–**2** and the free ligands (Chr, phen) (Figures S7 and S8). For that reason, four different cell lines were selected, corresponding to two different tissues, breast and lung, including both physiological and pathological (cancer) phenotypes, in a comprehensive time-, tissue-, and phenotype-dependent fashion for both new materials **1** and **2**. The results of the viability assay for 24, 48, and 72 h (Figure 12 and Figure 13) reveal significant impairment of the metabolic activity in the breast tissue cells, whereas only compound **2** reveals viability impairment in the lung tissue.

Specifically, the cancerous MCF7 cell line reveals atoxicity in DMSO solution (80–89% survival) compared to the control group, whereas 1% Triton X solution reveals significant cytotoxicity with 2–6% survival depending on the exposure time. With respect to hybrid material **1**, the survival is 61% at 24 h, increasing to ~82% at 48 h and 72 h, thereby revealing a significant adaptation period of the cells to the treatment environment. On the other hand, cell incubation with compound **2** reveals mild cytotoxicity, with the survival rate being 54–66% depending on the exposure time. A close look at the physiological breast cell line (MCF10A) reveals that DMSO incubation projects a significant adaptation period (48 h), while it is evident from the 72 h incubation that it is not cytotoxic, with a survival rate of 93%. The positive control treatment (1% Triton X) reveals significant cytotoxicity as expected (2.0–2.5% survival rate). Both compounds **1** and **2** are shown to be cytotoxic in this cell line, with compound **1** being mildly cytotoxic, exhibiting a 62% survival rate in 72 h, whereas the adaptation period of the cells reduces their metabolic activity over 24 h and 48 h, showing metabolic activity at 35% and 42%, respectively. On the other hand, compound **2** reveals increased cytotoxicity compared to **1**, with the survival rate at 41% in the 24 h exposure time and dropping further to less than 35% survival upon a 72 h incubation.

As for the ligand cytotoxicity, Chr at 10 μM reveals a slight cytotoxicity in the MCF7 cell line, with the survival rate being in the range 67–75% in all exposure times (24–48–72 h). Increasing the Chr concentration to 40 μM raises the cytotoxicity, revealing a reduced survival to 56–57% at 24 h and 48 h, and 42% at 72 h. Similarly, incubation of the cells with 40 μM phen exhibits significant time-dependent cytotoxicity, with the survival rates at 24 h and 48 h being 69% and 63%, respectively, whereas incubation for 72 h drops the survival rate to 49%. Finally, in the physiological MCF10A cell line, incubation with 10 μM Chr reveals slight cytotoxicity only in the 72 h phase (83% survival rate), with an adaptation period of 24 h, whereas at the same time, increasing the concentration to 40 μM increases cytotoxicity in the shorter incubation phases (83% in 48 h and 54% in 72 h), with the same adaptation period of 24 h as in the case of the 10 μM Chr. Finally, incubating MCF10A cells with 40 μM phen reveals significant cytotoxicity, with survival rates at 31% and 36% at 48 h and 72 h, respectively, whereas, for the 24 h viability reduction, that might be caused either by adaptation of the cells or cell cytotoxicity in this time period.

Moving to the lung tissue and, specifically, the physiological MRC-5 cell line, there is no significant impairment of the survival rate in any of the exposure times tested (24, 48, and 72 h) following incubation of the cells with DMSO, or compound **1**, where the positive control decreases the survival rate of the cells significantly (<3% in all exposure periods). Incubation with compound **2** shows mild cytotoxicity at 72 h, with the survival rate dropping to 71%, whereas significant impairment of the metabolic rate of the cells is observed at 48 h, likely due to the adaptation of the cells. In the case of the cancerous phenotype (A549), DMSO and compound **1** treatment reveals no impairment of the metabolic activity of the cells, whereas Triton X incubation projects significant cytotoxicity, with nearly 3% or less survival rate(s) in all exposure timeframes.

Regarding compound **2**, it shows cytotoxicity in a time-dependent fashion, with 80%, 67%, and 39% survival at 24, 48, and 72 h, respectively. As for the organic ligands, nearly none of the experimental conditions reveals cytotoxicity, except for the incubation with 40 μM phen for 72 h. It should be borne in mind that impairment of the metabolic activity observed in the case of 40 μM Chr at 24 h (nearly 71%) is likely due to the adaptation of the cells to the new conditions (addition of Chr). On the other hand, in the pathological A549 cell line, Chr incubation reveals an adaptation period for both concentrations tested, thus reducing the metabolic activity to 83–84% in both cases, and then restoring it at 48 h and 72 h. Furthermore, incubation of the cells with 40 μM phen reveals time-dependent cytotoxicity over 48 h and 72 h, with 77% and 54% survival rates, respectively, and a mild metabolic impairment (82%) in 24 h, with the adaptation period being more likely the cause of the observed phenotype, without definitively discounting cytotoxicity. It should be mentioned that, in all cell lines, the numerical mean values provided above bear a SEM < 12% for the MRC-5 cell line experiments (due to their increased metabolic activity) and less than 10% in all other cell lines.

Summarizing the viability results, it can be stated that breast tissue cell cultures reveal reduced viability of the physiological phenotype cell line (MCF10A), compared to the cancer cell line (MCF7), whereas, on the other hand, the physiological lung tissue cell line (MRC-5) has a greater adaptation period compared to the cancerous cell line (A549), with increased viability at the end of the incubation period (72 h). Interestingly, both physiological cell lines (MCF10A and MRC-5) have an adaptation period of nearly 48 h, under specific treatment conditions, during which they reveal reduced metabolic activity, with the MRC-5 case being more evident.

With respect to the cytotoxicity effects of the compounds, compared to the ligands contained in compounds **1** and **2** (Chr and phen), results differ between the different cell lines (Figures S7 and S8). To that end, in the MCF10A case, both compounds reveal enhanced cytotoxicity compared to the corresponding free ligand concentrations, whereas, in the cancer cell line (MCF7), compound **1** reveals reduced metabolic activity compared to the free Chrysin ligand, with compound **2** exhibiting increased viability compared to its corresponding free ligands. In both lung cancer cell lines, compound **1** has no effect on the viability of both the physiological and cancer cell lines, whereas compound **2** reveals increased viability impairment compared to the free Chrysin ligand and slightly increased compared to the phenanthroline ligand.

#### 2.12.2. Cell Morphology Studies

**Figure 14:**
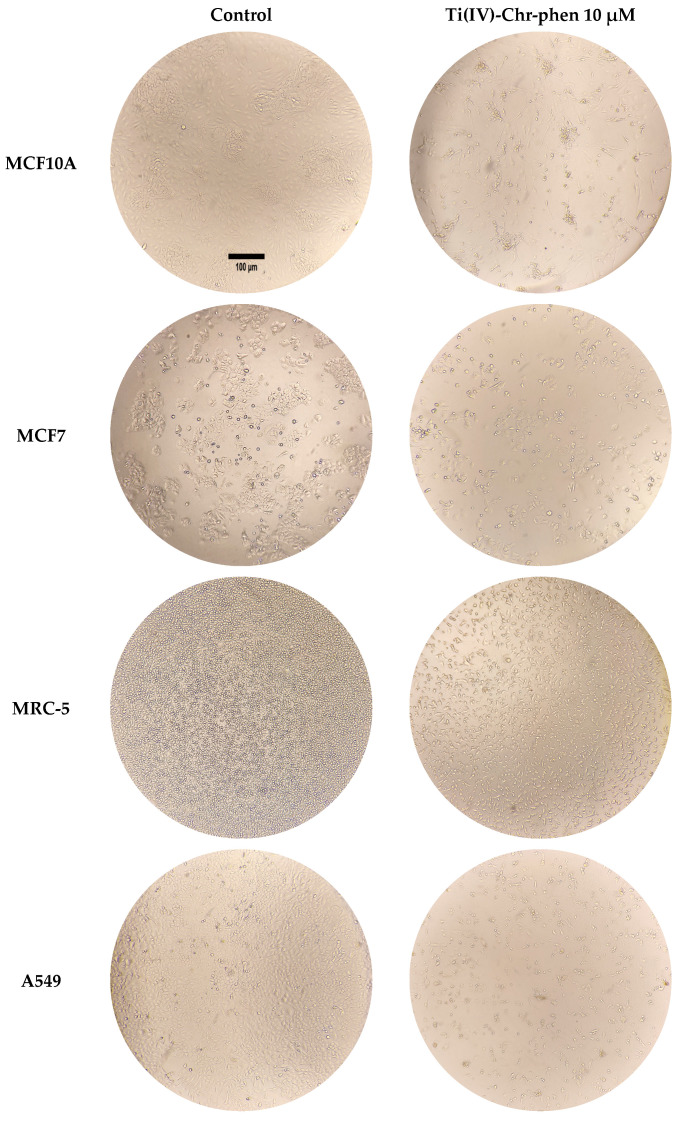
Cell morphology studies on MCF10A, MCF7, MRC-5, and A549 after incubation for 72 h with either complete medium (control) or 10 μM of Ti(IV)-Chr-phen.

To further assess the in vitro cytotoxicity of the newly synthesized materials **1** and **2** in a concentration-, time-, and cell line-dependent fashion, cell morphology assessment studies were carried out in all cell lines. The main morphological alterations, as shown in Figures S9–S12, emerge upon incubation with Ti-Chr-phen (**2**) at a concentration of 10 μM, and they become more profound after 72 h of incubation (Figure 14), with the corresponding incubation employing free phen exhibiting alterations in all cases. In addition, the only other significant morphology changes in the cell lines are observed upon incubation with Chr at a concentration of 40 μM in the MCF7 cell line, indicating enhanced cytotoxicity. On the other hand, the cell morphology alterations observed are not significant, leading to the assumption that the viability impairment picture observed merely reflects an adaptation period of the cells to the medium containing the corresponding compound or ligand (vide supra), a contention further validated through the delayed viability enhancement.

## 3. Discussion

### 3.1. Structural Speciation of Ti(IV)-Flavonoid Compounds

Metal–organic compounds are increasingly attracting the attention of the scientific community as promising biologically active materials in the field of treating heterogeneous diseases, such as cancer. It is well-known that many cases of cancer are linked to inflammation and mostly to chronic inflammation associated with oxidative stress. Addressing targeted therapies toward designated tumor tissues, without affecting healthy tissues and concurrently minimizing or eliminating side-effects, is a great challenge in the field of biomolecular engineering linked to the synthesis of new pharmaceutical agents bearing such properties. In that respect, in the herein presented study, titanium(IV) was selected as the metal center of purportedly bioactive new metalloforms bearing antioxidant flavonoids. Ti(IV) is a non-platinum metal ion, which has been shown to exhibit antitumor activity through different ways of biochemical reactivity [41].

It appears that Ti(IV) is isoelectronic to V(V) (3d^0^), which is plethorically undergoing synthetic and theoretical research work as a biologically important metal ion in cellular physiology and disease therapeutics. Past and ongoing work with vanadium and its biological potential in theranostics has been at the forefront of our lab’s research at the synthetic and biochemical level [42,43,44,45]. To that end, comparison of the two abutting metal ions (titanium and vanadium) at this juncture attracts considerable attention and signifies the importance of Ti(IV) choice, when delving into the corresponding chemistries, while concurrently exploring the biological potency of the derived metal–organic species. To that end, Ti(IV) would be expected to exhibit similarities to V(V) in chemical reactivity, which could provide insight into a new physicochemical investigation relevant to biological systems. Consequently, our approach includes the introduction of Ti(IV) into interactive chemistries with well-known antioxidant, anti-inflammatory, anticancer, anti-diabetic, etc., polyphenolic compounds (flavonoids) [46]. In this case, Chrysin (Chr) was selected as the flavonoid of choice, as it has been reported to exhibit inhibitory properties against tumor and tumor cell-induced angiogenesis [47]. Concurrently, there have been several research reports focusing on chemical modifications of Chr that might exhibit especially enhanced antitumor activity due to increasing solubility and bioavailability [48,49]. Therefore, it is of great importance to probe into structurally dictated metal binding modes of Chr and, therefore, its chemical reactivity toward Ti(IV), thus combining two important agents of great therapeutic potential. Consequently, the effort launched in our lab has focused on the chemical reactivity of (a) binary systems of Ti(IV) in the presence of Chr, and (b) ternary systems in the presence of an additional ancillary aromatic chelator phen, extensively perusing their synthetic efficacy toward well-defined and pure materials in a stoichiometric and temperature-dependent manner.

Synthetic investigations were carried out in alcoholic media investigating the aforementioned binary and ternary systems, with an optimized molar stoichiometry of Ti(IV):Chr 5:1 and Ti(IV):Chr:phen 1:1:1, respectively. In the case of the binary system, a mononuclear assembly was isolated [Ti(C_15_H_9_O_4_)Cl(CH_3_O)(CH_3_OH)_2_]Cl•CH_3_OH (compound **1**). In the case of the ternary system [Ti_4_O_4_(C_15_H_9_O_4_)_4_(C_12_H_8_N_2_)_4_]Cl_4_•2CH_3_OH• 5(CH_3_)_2_CHOH (compound **2**), a well-known chelating molecule was used (phen), leading to a tetranuclear cluster, and thereby showing that there is a strong competition of Chr and phen for chelation to the Ti(IV) center, in the presence of triethylamine (Et_3_N) assisting in the deprotonation of the phenolic C(5)-OH of the Chr ligand. The valence of the metal center in each complex assembly was verified through theoretical calculations (BVS) as Ti(IV) for both compounds. Using excess of TiCl_4_, only in the case of the binary system, seems to justify its property as a strong Lewis acid, thus being capable of contributing to the Ti(V)-O bond formation through deprotonation of the phenolic C(5)-OH and leading to a mononuclear structural assembly in **1**. The ternary system assembly in **2** seems to be amply facilitated through the employed high temperature reaction process. The overall chemical reactivity pathways investigated were optimally shown to be specific for every assembly, leading to discrete molecules. To investigate further the association between the binary and ternary systems, a transformation reaction was pursued. In fact, the binary system Ti(IV)-Chr (**1**) was subjected to chemical transformation reactivity in the presence of the aromatic phen chelator under solvothermal conditions. The emerging ternary crystalline product **2** substantiates the belief that there exist discrete species under the prevailing reaction conditions that could be isolated, and their biological properties investigated, thereby lending credence to the fact that the structural speciation of the specific systems is intimately linked to well-defined assemblies with discretely notable biological activity (vide infra).

### 3.2. Theoretical Interactions and Solid-Solution Properties

To further understand the properties of the structural assemblies of Ti(IV)-Chr (**1**) and Ti(IV)-Chr-phen (**2**) in the solid-state and in solution, the well-characterized and crystallographically defined materials were physicochemically probed into theoretically and experimentally (Hirschfeld surface analysis, FT-IR, 1D and 2D NMR, ESI-MS, Luminescence, and X-ray crystallography). In that respect, the binary and ternary systems were depicted in 3D color mapping through surface analysis, and the most abundant interactions were further quantified through 2D Fingerprint plots. The relevant analysis shows that the methanolic moiety bound to the mononuclear Ti(IV)-Chr assembly (**1**) is involved in strong O–H···O hydrogen bonds, whereas in the ternary compound, Ti(IV)-Chr-phen (**2**), the C(5)-phenolic group of Chr is involved in the same interactions, leading to H···O/O···H interactions totaling 10.2% and 11.0%, respectively. In addition, there are slight C–H···π interactions related to bound Chr of neighboring moieties in both assemblies (11.0% for the binary and 16.1% for the ternary material). Due to the more complex assembly of Ti(IV)-Chr (**1**), planar stacking arrangements are observed due to the presence of aromatic rings in Chr and phen ligands bound to Ti(IV). The overall H···H interactions, emerging from the presence of Chr, turn out to be at the highest level proportionally, reaching a value of 50.8% for Ti(IV)-Chr (**1**) and 55.6% Ti(IV)-Chr-phen (**2**). The collective interactions indicate that the bound methanolic moiety and the chloride ion are very crucial in supporting the binary system, whereas, in the ternary system, the C(5)–OH group possesses that specific role and is involved not only in the binding to the metal ion, but also in well-defined intermolecular and intramolecular interactions.

The presence of the Chr flavonoid bound to the metal center as well as the phen chelator was verified through FT-IR and the UV–Visible electronic patterns. Specifically, shifts related to the carbonyl group of the flavonoid are depicted in the FT-IR spectra, showing a shift of 19 cm^−1^ for **1** and 28 cm^−1^ for **2**, compared to that of free Chr. Also, a differentiation in the pattern is observed in the range ~3400–3500 cm^−1^, indicating deprotonation of Chr, which subsequently binds to Ti(IV). In the case of the ternary system in **2**, features specific to C-H and C-N vibrations, reflecting bonds in phen, attest to the incorporation of the aromatic chelator in the tetranuclear assembly. All of the aforementioned indications were further examined through UV–Visible spectroscopy in a concentration-dependent manner, providing ε values for both compounds on the basic absorbance features (Figure 6). Characteristic peaks related to the cinnamoyl and benzoyl systems of the Chr flavonoid appear to have been shifted. New absorption peaks were also shown to be present at 392 nm for Ti(IV)-Chr (**1**) and 395 nm for Ti(IV)-Chr-phen (**2**) related to Ligand to Metal Charge Transfer, and thus verifying binding of Chr to the central metal ion. All absorption peaks were fitted using the Savitzky–Golay algorithm, and it was verified that there are no artifacts in the studied spectral patterns. The unraveled overall patterns, emerging through FT-IR and UV–Visible spectroscopies, were subsequently employed in experiments for further verification of the existence of new metalloforms using ESI-MS and 1D-2D NMR. In the case of Ti(IV)-Chr (**1**), ^1^H-NMR spectroscopy confirms the disappearance of the C(5)-OH group (Figure 7) downfield and slight shifts in the other hydrogen types of Chr. Also shown is the incorporation of methanolic and methoxido moieties in the structure of the complex assembly. The ^13^C NMR pattern attests to the incorporation of methanolic and methoxido moieties as well as related carbons shifted downfield, thus verifying that Chr retains its structure and is bound to the central metal ion Ti(IV). In the case of the ternary system **2**, both ^1^H and ^13^C-NMR spectra show the incorporation of the same phenolic group of C(5) (Figure S4A,B), indicating deprotonation of the abutting C(5)-OH moiety. There is no solvent intensity in this case, verifying that only Ti(IV), Chr, and phen are incorporated into the assembly. 1D NMR analysis was followed by 2D spectra acquisition, using gradient correlation of ^1^H-^1^H (gCOSY) and ^1^H-^13^C (gHSQC) spectra. It is shown clearly that H6 and H8 (Figure 7 and Figure S4A) correlations of the Chr moiety are present, confirming a scalar coupling network within the A-ring skeleton post-coordination. Furthermore, all ^1^H-^13^C interactions related to the benzoyl ring show the expected interactions, thus verifying the retained structure of the Chr-bound moiety. All of the aforementioned physicochemical properties have been further verified through X-ray crystallography. Solid-state luminescence properties reveal quenching compared to that of free Chr and phen free molecules, thus confirming not only complexation to Ti(IV) as in our previous studies [21,22,28], but they also provide evidence of a strong interaction between the flavonoid and the metal ion. To that end, the experimental data on the studied metal–organic compounds support their potential future use in sensing or photochemical studies.

### 3.3. Antibacterial and Anti-Inflammatory Properties

The well-defined and well-characterized metal–organic materials **1** and **2** have been further investigated for their potential antibacterial properties. Two different strains have been employed, including Gram(−) (*Escherichia coli*) and Gram(+) (*Staphylococcus aureus*), through the disk diffusion method in solid-state bacterial cultures. A concentration-dependent study was thus carried out, leading to the determination of the MIC (expressed as mass amount and mg/cm^2^) values for **1** and **2**, the free ligands (Chr, phen), and TiO_2_. The experimental data show that Ti(IV)-Chr (**1**) exhibits an MIC value of 0.50 mg (ZOI 31.5 ± 0.1 mm) for *E. coli* and 10 mg (35 mg/cm^2^) (ZOI 13.5 ± 0.2 mm) for *S. aureus*. The ternary compound Ti(IV)-Chr-phen (**2**) exhibits an MIC value of 0.080 mg (0.28 mg/cm^2^) (ZOI 27.5 ± 0.1 mm) for *E. coli* and 10 mg (35 mg/cm^2^) (ZOI 15.3 ± 0.1 mm) for *S. aureus*. Chr alone shows no antibacterial effect in the range of concentrations studied, whereas the phen ligand shows a lower ZOI value than the ternary complex in *E. coli* and a higher ZOI value in *S. aureus* (almost double). As for TiO_2_, under the experimental conditions employed, it exhibits an antibacterial effect only in *E. coli*, thus confirming that, basically in the case of Ti(IV)-Chr-phen, the effect is related to the presence of Chr and phen moieties. As for the Ti(IV)-Chr system (**1**), the experimental evidence suggests that the molecular structural assembly is capable of inducing an antimicrobial effect against both strains. Moreover, the overall results attest to the enhancement of antibacterial properties of Chrysin through its coordination to Ti(IV). Based on the a) phenotypic behavior of the bacterial cells investigated, and the structural composition and assembly of the bacterial cell membranes, it is postulated that, due to the thicker cell wall membrane and thinner peptidoglycan layer of *E. coli* compared to that of *S. aureus*, both compounds appear to gain access to the inner cell space of the Gram(−) organisms at lower concentrations than in their Gram(+) counterparts. That way, the observed phenotype exemplifies the ability of the title materials to counteract bacterial infections linked to chronic inflammation processes or even bacterially induced inflammatory pathways linked to carcinogenesis [50,51,52]. However, regardless of the cause and effect relationships linking bacterial infections, inflammation, and cancer-associated processes, further investigation is needed to (dis)prove and delineate the pathways through which the activity of the title compounds avails itself in the experiments carried out.

Being cognizant of the fact that the extensively studied compounds exhibit enhanced antibacterial properties against Gram(+) and Gram(−) bacteria, both materials were further employed in anti-inflammatory studies. To that end, the anti-inflammatory activity of Ti(IV)-Chr (**1**) and Ti(IV)-Chr-phen (**2**) was studied extensively through the BSA denaturation assay. Protein denaturation is a well-known mechanism of inflammatory response, as structural damage leads to tissue inflammation. In the framework of the present study, the experimental results were compared to the well-known anti-inflammatory agent Diclofenac, and the final inhibition potential was expressed as IC_50_ values.

Both compounds exhibited significant inhibition of BSA denaturation in a concentration-dependent manner (0.001 μg/mL–20 μg/mL), with maximum inhibition observed at 0.364 ± 0.031 μg/mL in the case of Ti(IV)-Chr (**1**) and 0.160 ± 0.039 μg/mL in the case of Ti(IV)-Chr-phen (**2**). Compared to the free flavonoid ligand Chr (0.954 ± 0.07 μg/mL), the metal–organic compounds show enhanced inhibitory effects, suggesting that metal coordination contributes to the anti-inflammatory activity. This enhancement may be due to increased stability, better target protein interaction involved in (anti)inflammatory mechanistic pathways or synergistic antioxidant properties revealed by metal–flavonoid coordination. The emerging experimental results project activity ~10 times higher than the reference diclofenac agent (14.8 ± 1.3 μg/mL), thereby providing solid evidence that the two Ti(IV) compounds could suppress inflammation by stabilizing protein targets (e.g., cytokines) and interfering with denaturation-related inflammatory pathways.

### 3.4. In Vitro Biological Activity in Breast and Lung Tissues

The potency of the synthesized materials **1** and **2**, and their corresponding organic ligands were tested against cancerous cell lines from breast and lung tissue in comparison with the physiological substrates (present in the coordination sphere of **1** and **2**), using the XTT viability assay followed by morphology studies. In breast tissue cell cultures, it is evident that the viability of the physiological cell line (MCF10A) is impaired following treatment with both materials **1** and **2**. Comparison with the cancerous cell line (MCF7) reveals that the title compounds are more toxic to the physiological tissue than to cancer cells, thereby lending credence to the notion that the specific materials may not be specifically effective against breast tissue cancer (Figure 12). At the same time, comparison of the same compounds with the corresponding organic ligand substrates reveals that both materials exhibit similar viability impairment effects as the free ligands. The ensuing cell morphology studies (Figure 14) validate the results of cytotoxicity of the ternary Ti(IV)-Chr-phen and the corresponding phen ligand, whereas no significant morphological alteration is observed in the case of the binary Ti(IV)-Chr compound, thus indicating a different response from the specific cells.

Contrary to the above observations, in the lung tissue, long-term (72 h) incubation of the cell culture with the ternary compound **2** (Ti(IV)-Chr-phen) reveals significant differential viability between the two cell lines, favoring the survival of the physiological cell line (MRC-5). In the physiological cell line (MRC-5), it is evident that there is a significant adaptation period (nearly 48 h), attested to through a) the enhancement of viability in the 72 h incubation period, and b) the cell morphology examination results, where cell morphology changes are not significant (only an imperceptible reduction on the total confluency of the cells is observed) compared to the corresponding changes observed with the phen ligand, where cell morphology was significantly altered. In the case of the cancer cell line (A549), the ternary compound **2** causes greater cell viability reduction compared to the ligands alone, which is further validated by the cell morphology studies, where the phen ligand and the ternary compound reveal significant alterations in the structure and size of the A549 cells. In the lung tissue, the binary compound **2** (Ti(IV)-Chr) reveals no cytotoxicity under the experimental conditions tested. These results indicate that the ternary compound **2** (Ti(IV)-Chr-phen) is potent against lung tissue cancer, when results are compared between the pathological and the physiological cell lines, and validated through viability and cell morphology studies extended up to 72 h. Further consideration of interactions of the species with (sub)cellular targets, due to which the phenotypic behavior manifests in the tissue cultures investigated in vitro, could be attributed to the biodistribution of the soluble and bioavailable titanoforms throughout the biofluid contents of the cells. Such potential targets involve a plethora of molecules (including phosphorylated and non-phosphorylated proteins and enzymes, as well as genetic loci linked to metabolic and signaling pathways) [53,54,55,56,57], the degree of specificity and extent of which exemplifies the observed biochemical behavior and is currently under scrutiny in the lab.

## 4. Materials and Methods

### 4.1. Reagents and General Procedures

Titanium(IV) chloride (TiCl_4_) was purchased from Fluka (Honeywell International Inc., Charlotte, NC, USA). The flavonoid Chrysin (C_15_H_10_O_4_, >98.0%, Chr) was purchased from TCI (Tokyo Chemical Industry CO., Ltd., Tokyo, Japan). 1,10 Phenanthroline (C_12_H_8_N_2_, 99.0%, phen) and appropriate solvents used in the synthetic chemistry reactivity and analytical measurements (Methanol, 2-Isopropanol, and Dimethyl sulfoxide ((CH_3_)_2_SO, DMSO)) were acquired from Sigma Aldrich (Sigma-Aldrich Chemical Company, Steinheim, Germany). Furthermore, triethylamine (C_6_H_15_N) was provided by Carlo Erba (Carlo Erba Reagents GmbH, Emmendingen, Germany). LC-MS grade methanol (99.9%) was purchased from Chem-Lab (Chem-lab NV, Zedelgem, Belgium).

**Note:** Titanium(IV)-chloride was handled under a fume hood, using Argon 99.9999% to provide an inert atmosphere during the synthesis of the title compounds. Specifically, glassware and solvents in the reactions run were maintained under an inert gas (Argon) atmosphere on a Schlenk line to avoid moisture affecting the primary reagent.

### 4.2. Physical Measurements

Quantitative determination of carbon, hydrogen, and nitrogen was pursued using a ThermoFinnigan Flash EA 1112 CHNS elemental analyzer (Thermo Fisher scientific Inc., Waltham, MA, USA) with a dynamic flash combustion of samples at 1800 °C. Further rapid determination of the products was evaluated through reduction, trapping, complete GC separation, and detection in a fully automated manner and controlled by PC via the Eager 300 dedicated software system.

FT-Infrared spectra were recorded on a Nicolet FT-IR 200 spectrometer (Thermo Fisher Scientific Inc., Waltham, MA, USA), using KBr pellets, at room temperature conditions.

UV–Visible spectroscopy measurements of all solutions prepared were carried out on a Hitachi U-1900 spectrophotometer (Hitachi Ltd., Tokyo, Japan), in the range 190–1100 nm. The SYSTAT Inc. peakfit (v 4.11) program was employed for the spectral fitting of the Ti(IV) hybrid metal–organic compound solution spectra. Savitzky–Golay algorithms were employed for the fitting process, using (a) number of iterations 8000–10,000, (b) number of significant digits 6, and (c) full curvature matrix, until R^2^ indicates a value of 0.9999 ± 0.0001.

#### 4.2.1. ESI-MS Spectrometry

A Thermo Fisher Scientific model LTQ Orbitrap Discovery mass spectrometer was employed for spectrometric measurements on compounds **1** and **2**, along with Electrospray ionization (ESI-MS) (Bremen, Germany) in LC-MS grade methanol. Solutions of compounds **1** and **2** with molecular formulae M1 = [Ti(C_15_H_9_O_4_)Cl(CH_3_O)(CH_3_OH)_2_]^+^ = C_18_H_20_TiClO_7_ and M2 = [Ti_4_O_4_(C_15_H_9_O_4_)_4_(C_12_H_8_N_2_)_4_]^4+^ = C_108_H_68_Ti_4_N_8_O_20_ were introduced into the ESI source of the MS at a flow rate of 5 μL/min, using an integrated syringe pump. The infusion experiments were run using a standard ESI source, operating in a positive ionization mode. Source operating conditions were as follows: 3.7 kV spray voltage and 320 °C heated capillary temperature.

#### 4.2.2. Solution ^1^H-, ^13^C-NMR and 2D NMR

Nuclear Magnetic Resonance Spectroscopy experiments for **1** and **2** were carried out on a Varian 600 MHz spectrometer (Agilent, Germany) in the solution state, using DMSO-d^6^ (Sigma-Aldrich chemical company, Steinheim, Germany) at 298 K. 1D and 2D spectra were recorded at a sample concentration of 10 mM, using 3-(Trimethylsilyl)-propionic-2,2,3,3-d^4^ acid sodium salt (TSP) (Sigma-Aldrich chemical company, Steinheim, Germany) as internal standard. Proton (^1^H) spectra were acquired with 512 transients and a spectral width of 5000 Hz. Carbon (^13^C) spectra were acquired with 5000 transients, a spectral width of 37,000 Hz, and a relaxation delay of 5 s. ^1^H PRESAT was conducted using 8 scans and a relaxation delay of 2 s through a two-step purge experiment. 2D spectra gCOSY and gHSQC were recorded using 1s relaxation delay and 400 t1 increments in the ^13^C dimension. Experimental data were processed using VNMR routines. Spectra were zero-filled and subjected to exponential apodization before Fourier Transformation (FT). Chemical shifts (δ) are reported in ppm, and the overall spectra were analyzed through the Mnova software 14.2.1 (Mestrelab Research S.L., Santiago de Compostela, Spain).

#### 4.2.3. Photoluminescence

Solid-state luminescence activity of compounds **1**–**2** was investigated on a Hitachi F-7000 fluorescence spectrophotometer from Hitachi High-Technologies Corporation (Hitachi Ltd., Japan) at room temperature conditions. Appropriate solid-state spectra (Emission (em) and excitation (ex)) were recorded using split widths of 5.0 nm and a scan speed of 1200 nm min^−1^. The entire system was supported by Windows XP, and it employed the FL Solutions 2.1 software.

### 4.3. Bond Valence Sum

Bond Valence Sum (BVS) calculations for the Ti(IV) metal centers were carried out using the Visualization for Electronic and Structural Analysis program (VESTA, Version 3.5.8). The empirical expression was used for calculations (S = exp[(Ro − r)/b]), with b set at 0.37 Å and the tabulated constant (Ro) set to 1.815 Å for Ti(IV)-O [58].

### 4.4. Hirshfeld Surface Investigation

Hirshfeld surface analysis of **1**–**2** was generated using Crystal Explorer (University of Western Australia, Crawley, Australia) [59] and Inkscape 1.0.1 program (Inkscape, Boston, MA, USA). 3D mapping of the surfaces (d_norm_, shape-index and curvedness) [60,61,62] as well as two-dimensional (2D) Fingerprint plots (d_e_ vs. d_i_) [63,64,65] were employed for the interpretation of various intermolecular and intramolecular interactions. During mapping, bond lengths to hydrogen atoms were set to standard values [66].

### 4.5. Synthesis

**Synthesis of Ti(IV)-Chr, [Ti(C_15_H_9_O_4_)Cl(CH_3_O)(CH_3_OH)_2_]Cl•CH_3_OH (1)**. In a 50 mL round-bottom flask, 10 mL of methanol was added and was further cooled down for 30 min using an ice bath. To that, 0.50 mL (5.0 mmol) of TiCl_4_ was added under argon in a fume hood. The following clear light-yellow solution was allowed to return to room temperature for 40 min. Subsequently, Chr (0.25 g, 1.0 mmol) was added under continuous stirring, resulting in a clear dark orange solution. After 1 h of stirring, the final reaction mixture was filtered and allowed to stand at room temperature. Five days later, prismatic orange crystals were isolated by filtration. Yield 0.42g (18 ± 1%). Anal. Calcd. for **1**, (C_19_H_24_Cl_2_O_8_Ti M_r_ = 499.20): C, 45.69; H, 4.81. Found: C, 45.60; H, 4.79.

**Synthesis of Ti(IV)-Chr-phen, [Ti_4_O_4_(C_15_H_9_O_4_)_4_(C_12_H_8_N_2_)_4_]Cl_4_•2CH_3_OH• 5(CH_3_)_2_CHOH (2)**. In a 50 mL round-bottom flask, containing 10 mL of a cold mixture of methanol/2-propanol (50% *v*/*v*), TiCl_4_ (0.05 mL, 0.50 mmol) was added under continuous stirring. The reaction mixture turned clear and reached room temperature after 4 min. Subsequently, 0.13 g (0.50 mmol) of Chr were added under stirring. The new reaction solution turned cloudy yellow. Subsequently, phen (0.090 g, 0.50 mmol) was added and the solution remained unchanged. Addition of triethylamine (0.07 mL, 0.50 mmol) resulted in a cloudy orange solution mixture. The final reaction mixture was transferred to a Teflon-lined stainless-steel reactor (23 mL) and heated at 90 °C for 17 h. Gradual cooling of the reaction led to orange bar crystals, which were isolated by filtration. Yield: 0.13 g (53 ± 2%) Anal. Calcd. for **2** (C_125_H_116_Cl_4_N_8_O_27_Ti_4_ M_r_ = 2495.74): C, 60.10; H, 4.64; N, 4.49. Found: C, 59.98; H, 4.62; N, 4.52.

**Transformation of Ti(IV)-Chr (1) to Ti(IV)-Chr-phen (2)**. In a 50 mL round-bottom flask, containing 10 mL of a mixture of methanol/2-propanol (50% *v*/*v*), 0.20 g (0.40 mmol) of Ti(IV)-Chr (1) was dissolved and continuously stirred for 30 min, ultimately giving rise to a slightly cloudy orange solution. Subsequently, 0.070 g (0.40 mmol) of phen and 0.060 mL (0.40 mmol) of triethylamine were added under continuous stirring, and the emerging reaction mixture turned cloudy dark orange in color. The final reaction mixture was transferred to a Teflon-lined stainless-steel reactor (23 mL) and heated at 90 °C for 17 h. Once the reaction mixture was cooled down to room temperature, orange bar crystals emerged that were isolated by filtration. Yield: 0.095 g (38 ± 1%). The material was further identified as **2** through FT-IR spectroscopy and X-Ray Crystallography.

### 4.6. X-Ray Structural Determination

Suitable single crystals of **1** and **2** were analyzed through single-crystal X-ray diffraction. The single crystals were mounted on a Bruker Kappa APEX II X-ray diffractometer (Bruker Analytical X-ray Systems, Inc. Madison, WI, USA), equipped with a TRIUMPH monochromator. Radiation from a Mo source was used to record diffraction measurements at room temperature. A minimum of 120 reflections in the range of 15 < θ < 20° were employed to accomplish refinement of cell dimensions, while φ and ω scan modes were used to collect intensity data. Bruker SAINT (Bruker Analytical X-ray Systems, Inc. Apex2, Version 2 User Manual, M86-E01078, Madison, WI, USA, 2006) software was used to process the reflection data for each crystal via a narrow-frame algorithm [67]. The SADABS numerical method, based on the dimensions of the crystals, was used to correct the data for absorption [68]. Both structures were solved by charge-flipping methods, implemented in Superflip [69], and refined by a full matrix least-squares procedure based on F^2^, using CRYSTALS software 14.61_build_6236 [70]. Asymmetric units in both crystals contain a cationic main complex, chloride counter anions, and/or methanol and isopropanol solvate molecules. Non-hydrogen atoms in both main complexes were non-disordered and refined anisotropically. Solvate molecules in complex **2** were found disordered over two or even more positions. All non-hydrogen, non-disordered atoms were refined anisotropically. For the disordered atoms, their occupancy factors and positions under fixed isotropic parameters (U_iso_ = 0.05) were first refined. After this step, they were all refined isotropically under fixed occupancy factors. Hydrogen atoms riding on parent non-disordered atoms were located on their expected positions and refined with isotropic displacement parameters U_iso_(H) = 1.2U_eq_(C) or 1.5U_eq_ for -OH hydrogens and at distances C--H 0.95 Å and O-H 0.82 Å. All methyl and OH hydrogen atoms were allowed to rotate. Hydrogen atoms, riding on disordered oxygen atoms of methanol and isopropanol solvent molecules, were positioned geometrically to fulfill hydrogen-bonding demands. The remainder of the methyl hydrogen atoms were positioned geometrically with respect to their parent atoms.

### 4.7. Antibacterial Properties In Vitro

The disk diffusion method was pursued on autoclavable 25 mL Luria–Bertani agar (LB agar) (Applichem PanReac, Darmstadt, Germany) Petri dishes of 90 mm in diameter [71]. Disk samples (6 mm), containing the hybrid metal–organic compound and LB Agar mixtures at different concentrations, with a total testing material mass of 30 mg, were employed. Minimum Inhibitory Concentration (MIC) (expressed as mass amount (mg) and mg/cm^2^ (mg/area of studied disk = mg/0.28 cm^2^)) values were determined in Gram-positive (Gram(+)) (Staphylococcus aureus; *S. aureus*) and Gram-negative (Gram(−)) (Escherichia coli; *E. coli*) bacterial cultures though appropriate measurement of standard Zone Of Inhibition (ZOI) following incubation for 12–15 h at 37 °C [72]. The Luria–Bertani broth (LB broth) (Sigma Aldrich, Munich, Germany) was also used for the production of the inoculum and the positive control (penicillin-streptomycin (Biowest, Nuaillé, France)). More specifically, bacteria were grown in 3 mL LB broth, using a 25 mL Erlenmeyer flask in an Edmund Bühler TH15 shaking incubator (Edmund Bühler Gmb, Bodelshausen, Germany) at 37 °C for 1–2 h, until the O.D. at 600 nm reached a value of 0.5, corresponding to the exponential phase of the growth curve (5 × 10^5^ CFU•mL^−1^). Optical density (O.D.) measurements, monitoring the growth of liquid cultures of bacteria, were carried out on a Hitachi UV–Visible U-2800 spectrophotometer (Hitachi, Tokyo, Japan). All experiments were run in triplicate under aseptic conditions.

### 4.8. Anti-Inflammatory Properties

The ability of hybrid metal–organic compounds to inhibit the heat-induced Bovine Serum Albumin (BSA) denaturation was investigated through the BSA denaturation assay [73,74,75,76]. Specifically, in a 96-well plate, 100 μL of BSA (Merck, Darmstadt, Germany) solution (0.4% w/v) in Phosphate Buffer Saline (PBS, 25.9 mM Na_2_HPO_4_, 1.78 mM KH_2_PO_4_, 2.74 mM KCl, 137 mM NaCl, pH 5.4) was mixed with 10 μL of sample solution, containing different concentrations of compounds in DMSO (0.001–20 μg/mL). The final volume was adjusted to 200 μL by addition of 90 μL PBS solution (pH 5.4). The plate was further incubated for 30 min at 37 °C and the turbidity of the mixtures was recorded at 595 nm, using a BioTek Synergy H1 Multimode Reader (Agilent Technologies, Santa Clara, CA, USA) at time intervals starting with t = 30 min after incubation. A mixture of 100 μL BSA solution (0.4% w/v) in PBS with 10 μL of DMSO and 90 μL of PBS was used as a control. Diclofenac sodium salt (Fagron Hellas, Trikala, Greece) was used, instead of the investigated sample compounds, as a reference sample in the concentration range 1–80 μg/mL in DMSO. The capacity of tested compounds to inhibit denaturation of BSA was calculated through Equation (1) [77], where A_c_ at t = 0 min is the absorbance of the control, and A_s_ at t = 30 min is the absorbance of every sample. The estimated Inhibitory Concentration values (IC_50_) for each compound were calculated through linear regression and were compared with the IC_50_ value of the reference anti-inflammatory drug (Diclofenac). All experiments were run in triplicates.

(1) $$\%\;Denaturation\;Inhibition=1-\frac{A_{s\;t=30}-A_{c\;t=0}}{A_{c\;t=30}-A_{c\;t=0}}\times 100$$

### 4.9. In Vitro Toxicity Profile Through Physiological and Cancer Cell Lines

#### 4.9.1. Cell Cultures

In the present investigation of the newly synthesized Ti(IV)-Chr-based materials **1** and **2**, cytotoxicity studies were carried out on four different human cell lines, representing two different tissues, i.e., (a) breast and (b) lung. In each tissue, a physiological cell line was evaluated simultaneously with the corresponding cancer cell line, with the experiments on cytotoxicity conducted in a time-, tissue-, and phenotype-dependent fashion for both materials. The selection of human physiological and cancer cell lines includes the following: MCF10A, an epithelial fibrocystic breast cell line isolated from the mammary gland; MCF7, a human epithelial breast adenocarcinoma cell line isolated from the same gland; MRC-5, a normal lung fibroblast cell line; and A549, an epithelial adenocarcinoma cell line, corresponding to physiological and cancer cell lines, respectively. Cells were seeded in 75 cm^2^ cell culture flasks and incubated under appropriate conditions (5% CO_2_ at 37 °C and humidified incubator) in Dulbecco’s modified Eagle’s medium DMEM (Biowest, Nuaillé, France), supplemented with 10% Fetal Bovine Serum FBS (Biowest, Nuaillé, France) and 1% penicillin-streptomycin (Biowest, Nuaillé, France). All experiments were run in low passages. To detach the cells, trypsin was added to the serum-free cell monolayer, and the plate was incubated at 37 °C. Subsequently, complete medium was added to inactivate trypsin, and the resulting mixture was centrifuged. The cell pellet was resuspended in complete medium, and the concentration of viable cells was determined using the trypan blue exclusion assay.

#### 4.9.2. Cell Viability Assay

Titanium compounds **1** and **2**, as well as controls (ligands), were used to investigate the potential effect(s) on cell viability and cell growth, according to the literature [78]. Specifically, 100 μL of cell suspension in complete medium was seeded in 96-multi-well plates (5000 cells/well). Following an overnight attachment period, the cultures were treated with the title materials for 24, 48, and 72 h in 1% *v*/*v* DMSO culture medium, using 10 μM of the title compounds, the corresponding 10 and 40 μM of Chrysin, and 40 μM of 1,10-phenanthroline. Cell viability/proliferation was assessed through the XTT assay. More specifically, once the timeframe of the experiment was completed, 50 μL of XTT detection solution (containing 49 μL of XTT reagent and 1 μL of electron coupling solution) was added in each well, and the plate was incubated in 37 °C for 4 h. Subsequently, the absorbance (A) of each well was measured at 450 nm, using a BioTek Synergy H1 Multimode Reader (Agilent Technologies, Santa Clara, CA, USA). The survival percentage was calculated by the following equation (Equation (2)):

(2) $$\%\;Viability=100\times \frac{A_{450\left(EC\right)}-A_{450\left(CM\;no\;cells\right)}}{A_{450\left(CM\;with\;cells\right)}-A_{450\left(CM\;no\;cells\right)}}$$

with EC standing for Experimental Condition and CM for Culture Medium. All of the experiments were performed in triplicates.

#### 4.9.3. Cell Morphology

Cell morphology was also studied in the presence of the title materials to further probe into potential cytotoxic effects, as mentioned previously [61]. To that end, 200,000 cells in 2 mL of proper medium were seeded per well of a six-well tissue culture plate, and the plate was incubated overnight to ensure attachment of the cells on the plate surface. Then, the medium was replaced by 2 mL of solution containing the studied materials in 1% *v*/*v* DMSO culture medium, using 10 μM of the title compounds, the corresponding 10 and 40 μM of Chrysin, and 40 μM of 1,10-phenanthroline. Cells were visualized, prior to treatment and post-treatment, at 0, 24, 48, and 72 h, using an Oxion Inverso biological microscope (Euromex, Duiven, The Netherlands) bearing 10× and 40× lenses.

### 4.10. Statistical Analysis

All obtained experimental data are presented as average±SEM values of multiple sets of independent measurements, followed by post hoc analysis (Tukey) using GraphPad Prism v.6 (GraphPad Software Inc., Boston, MA, USA). IC_50_ values of the compounds, pertaining to their anti-inflammatory properties, were calculated using log transformation of the concentration values, normalization of the absorbance, and, subsequently, non-linear regression modeling followed by post hoc analysis (Tukey) (vide infra). For in vitro biological assays, data were presented as average and standard error mean (SEM) values of triplicate sets of independent measurements. Mean survival rates, SEMs, and EC_50_ were calculated for each group. Absolute survival rates were calculated for each control group and one-way analysis of variance (ANOVA) was performed for all pair comparisons, followed by post hoc analyses (Tukey). Degrees of significance for all studies were assessed by three different rating values: * *p* < 0.05 (significant), ** *p* < 0.01 (highly significant), *** *p* < 0.001 (extremely significant), and **** *p* ≤ 0.0001 (extremely significant) or non-significant (*p* > 0.05).

## 5. Conclusions

Driven by the need to develop hybrid inorganic–organic complex materials, as alternative metallodrugs, capable of exerting biological activity on bacterial cultures as well as eukaryotic cell tissues of defined (patho)physiology, two novel binary and ternary Ti(IV) compounds **1** and **2** were synthesized and isolated as crystalline materials containing the flavonoid Chrysin (**1,2**) and the N,N’-aromatic chelator phenanthroline (**2**). The pursued synthetic chemical reactivity was further looked into through investigation of chemical conversion of the binary compound **1** to the ternary compound **2**, thus exemplifying the interconnection of the derived materials and the importance of structural speciation as a general approach to the synthesis of well-defined materials in seeking further evaluation for potential alternatives of metallodrugs against various pathologies. The physicochemical characterization of the new compounds formulated a comprehensive chemical profile, which was described through analytical, spectroscopic, and crystallographic work, further validated through theoretical Hirshfeld analysis and BVS calculations. The configured profile justified subsequent employment of **1** and **2** in in vitro biological testing in both bacterial as well as eukaryotic cultures. The emerging properties a) exemplified the observed biological activity, intimately linked to the nature of the two coordination complex materials, and b) were distinctly differentiated from each other through the introduction of the N,N’-aromatic chelator phen in **2**. The observed bioactivity behavior was quite discrete for Gram(+) vs. Gram(−) bacteria through the distinct MIC and ZOI values, thereby associating the uniquely achieved physicochemical identity of **1** and **2** with the specific structure and integrity of bacteria of the two classes. In a surprising turn, the anti-inflammatory and anticancer activity profile of the two compounds was quite distinct in the two different tissues employed, representing in vitro different pathological phenotypes in comparison to the corresponding physiological cell lines. The so-observed collective activity profile denotes specificity toward defined cancer tissues, thus suggesting that the nature of the investigated complex species plays a significant role in the interactions involved with the target cells presented to them. The nature and properties of the two materials reflect upon their bifunctional character in confronting concurrently both bacterial assaults as well as inflammatory events on different cell tissues in a uniquely defined fashion. To that end, they project merit toward further perusal of their (bio)chemical profile as potential multifunctional metallodrugs in bacterial and human (patho)physiology. Detailed studies delving into mechanistic details of the observed bioactivity for the individual compounds **1** and **2** are currently ongoing in our lab.

## Figures and Tables

**Table 1:** Data collection and experimental details on compounds **1** and **2**.

Compound	1	2
Chemical formula	C_19_H_24_Cl_2_O_8_Ti	C_125_H_116_Cl_4_N_8_O_27_Ti_4_
*M*_r_	499.20	2495.74
Crystal system, space group	Monoclinic *P*2_1_/*n*	Tetragonal *P*4_2_/*n*
Temperature (K)	295	295
*a* (Å) *b* (Å) *c* (Å)	7.5536 (6) 13.4847 (10) 22.4709 (17)	22.231 (6) 22.231 (6) 13.361 (5)
β (°)	90.322 (3)	90
*V* (Å^3^)	2288.8 (3)	6603 (4)
*Z*	4	2
Radiation type	Mo *K*α	Mo *K*α
µ (mm^−1^)	0.65	0.39
Crystal size (mm)	0.19 × 0.18 × 0.12	0.16 × 0.04 × 0.03
Data collection		
Diffractometer	Bruker Kappa Apex2	Bruker Kappa Apex2
Absorption correction	Numerical	Numerical
*T*_min_, *T*_max_	0.89, 0.93	0.98, 0.99
No. of reflections measured independent observed [*I* > 2.0σ(*I*)]	19,253 4344 3556	33,909 6381 3889
*R*_int_	0.019	0.036
(sin θ/λ)_max_ (Å^−1^)	0.612	0.615
Refinement		
*R*[*F*^2^ > 2σ(*F*^2^)] *wR*(*F*^2^) *S*	0.034 0.053 1.00	0.056 0.104 1.00
No. of reflections	3556	3889
No. of parameters	271	381
No. of restraints	-	11
H-atom treatment	H-atom parameters constrained	
Δρ_max_, Δρ_min_ (e Å^−3^)	0.26, −0.25	0.72, −0.42

**Table 2:** Interatomic distances (Å) and bond angles (^o^) in **1** and **2**.

	1		2	
**Distances (** **Å)**	Ti(1)—Cl(1)	2.2957 (8)	Ti(1)—O(3) ^i^	1.804 (3)
	Ti(1)—O(1)	2.0351 (14)	Ti(1)—O(1)	2.087 (3)
	Ti(1)—O(2)	1.8638 (15)	Ti(1)—O(2)	1.889 (2)
	Ti(1)—O(5)	1.7397 (15)	Ti(1)—O(3)	1.809 (2)
	Ti(1)—O(6)	2.1005 (17)	Ti(1)—N(1)	2.234 (3)
	Ti(1)—O(7)	2.0628 (15)	Ti(1)—N(2)	2.183 (3)
**Bond angles (^o^)**	Cl(1)—Ti(1)—O(1)	91.49 (5)	O(3) ^i^—Ti(1)—O(1)	172.47 (11)
	Cl(1)—Ti(1)—O(2)	95.19 (6)	O(3) ^i^—Ti(1)—O(2)	98.00 (11)
	O(1)—Ti(1)—O(2)	86.56 (6)	O(1)—Ti(1)—O(2)	86.25 (11)
	Cl(1)—Ti(1)—O(5)	98.91 (6)	O(3) ^i^—Ti(1)—O(3)	100.17 (15)
	O(1)—Ti(1)—O(5)	169.56 (7)	O(1)—Ti(1)—O(3)	85.04 (11)
	O(2)—Ti(1)—O(5)	93.31 (7)	O(2)—Ti(1)—O(3)	100.88 (11)
	Cl(1)—Ti(1)—O(6)	168.31 (5)	O(3) ^i^—Ti(1)—N(1)	88.30 (11)
	O(1)—Ti(1)—O(6)	79.20 (7)	O(1)—Ti(1)—N(1)	85.20 (11)
	O(2)—Ti(1)—O(6)	91.33 (7)	O(2)—Ti(1)—N(1)	93.84 (12)
	O(5)—Ti(1)—O(6)	90.37 (7)	O(3)—Ti(1)—N(1)	161.74 (11)
	Cl(1)—Ti(1)—O(7)	88.84 (5)	O(3) ^i^—Ti(1)—N(2)	92.02 (11)
	O(1)—Ti(1)—O(7)	86.09 (6)	O(1)—Ti(1)—N(2)	82.43 (11)
	O(2)—Ti(1)—O(7)	171.71 (7)	O(2)—Ti(1)—N(2)	163.06 (12)
	O(5)—Ti(1)—O(7)	93.22 (7)	O(3)—Ti(1)—N(2)	90.68 (11)
	O(6)—Ti(1)—O(7)	83.53 (7)	N(1)—Ti(1)—N(2)	72.74 (11)
			Ti(1)—O(3)—Ti(1) ^ii^	162.50 (14)

Symmetry codes: (i) −*y* + 1/2, *x*, −*z* + 1/2; (ii) *y*, −*x* + 1/2, −*z* + 1/2.

**Table 3:** ZOI values for **1**–**2** in Gram(−) (*E. coli*) and Gram(+) (*S. aureus*) bacteria.

		**Mass Content (mg)/Mass Content/Unit Area (mg/cm^2^)** **ZOI (mm)**		**Mass Content (mg)/Mass Content/Unit Area (mg/cm^2^)** **ZOI (mm)**
*E. coli*	Ti(IV)-Chr	0.50 mg (1.8 mg/cm^2^) 31.5 ± 0.1	Ti(IV)-Chr-phen	0.080 mg (0.28 mg/cm^2^) 27.5 ± 0.1
	TiO_2_	0.080 mg (0.28 mg/cm^2^) 29.7 ± 0.2	TiO_2_	0.010 mg (0.035 mg/cm^2^) 26.5 ± 0.1
	Chr	0.25 mg (0.88 mg/cm^2^) n.e.z.	Chr	0.040 mg (0.14 mg/cm^2^) n.e.z.
			phen	0.030 mg (0.11 mg/cm^2^) 20.5 ± 0.3
				
		**Mass Content (mg)/Mass Content/Unit Area (mg/cm^2^)** **ZOI (mm)**		**Mass Content (mg)/Mass Content/Unit Area (mg/cm^2^)** **ZOI (mm)**
*S. aureus*	Ti(IV)-Chr	10 mg (35 mg/cm^2^) 13.5 ± 0.2	Ti(IV)-Chr-phen	10 mg (35 mg/cm^2^) 15.3 ± 0.1
	TiO_2_	1.5 mg (5.3 mg/cm^2^) n.e.z.	TiO_2_	1.5 mg (5.3 mg/cm^2^) n.e.z.
	Chr	5.0 mg (17 mg/cm^2^) n.e.z.	Chr	4.6 mg (16 mg/cm^2^) n.e.z.
			phen	3.3 mg (12 mg/cm^2^) 30.2 ± 0.2

**Note:** n.e.z: no evaluable zone. 1% Penicillin/Streptomycin in *E. coli* show ZOI of 19.8 ± 0.2 mm. 0.05% Penicillin/Streptomycin in *S. aureus* show ZOI of 28.8 ± 0.5 mm.

## Data Availability

CCDC 2,473,594 (1) and CCDC 2,473,595 (2) contain the supplementary crystallographic data for this paper. These data can be obtained free of charge from the Cambridge Crystallographic Data Centre, 12 Union Road, Cambridge CB21EZ, UK; fax: +44-1223-336-033; or deposit@ccde.cam.ac.uk.

## Supplementary Materials

The following supporting information can be downloaded at: https://www.mdpi.com/article/10.3390/molecules30183667/s1, Figure S1. 2D Fingerprint of H^…^Cl/Cl^…^H interactions for **Ti(IV)-Chr** (**1**); Figure S2. (A) UV-Visible spectra of **2** compared to Chr and phen in methanol at 8 × 10^−6^ M; (B) Fitting spectra of **2**.; Figure S3. ESI-MS species detected in methanolic solution of (A) **1**; (B) **2**; Figure S4. (A) ^1^H NMR spectra of **2**; (B) ^13^C NMR spectra of **2**; recorded in DMSO-d^6^; Figure S5. (A) ^1^H-^1^H correlation through gCOSY NMR of **2**; (B) ^1^H-^13^C correlation through gHSQC NMR of **2**; Figure S6. Solid-state normalized luminescence spectra of **2** compared to (A) Chr; (B) phen; Figure S7. Viability assessment for Chr and phen, in physiological (MCF10A) and cancer (MCF7) human lung tissue cell cultures. Significance levels were assessed as follows: * *p* < 0.05 (significant), ** *p* < 0.01 (highly significant), *** *p* < 0.001 (extremely significant) and **** *p* ≤ 0.0001 (extremely significant); Figure S8. Viability assessment for Chr and phen, in physiological (MRC-5) and cancer (A549) human lung tissue cell cultures. Significance levels were assessed as follows: * *p* < 0.05 (significant), ** *p* < 0.01 (highly significant), *** *p* < 0.001 (extremely significant) and **** *p* ≤ 0.0001 (extremely significant); Figure S9. Cell morphology evaluation of MCF10A cultures after treatment with compounds **1** and **2**, and the corresponding ligands for 24, 48 and 72 h; Figure S10. Cell Morphology evaluation of MCF7 cultures after treatment with compounds **1** and **2**, and the corresponding ligands for 24, 48 and 72 h; Figure S11. Cell morphological evaluation of MRC-5 cultures after treatment with compounds **1** and **2**, and the corresponding ligands for 24, 48 and 72 h; Figure S12. Cell morphology evaluation of A549 cultures after treatment with compounds **1** and **2**, and the corresponding ligands for 24, 48 and 72 h; Table S1. Absolute error calculations based on m/z values of ESI-MS spectrometric measurements; Table S2. ^1^H-NMR shifts in DMSO-d^6^, 600 MHz; Table S3. ^13^C-NMR shifts in DMSO-d^6^, 600 MHz.

## Abbreviations

The following abbreviations are used in this manuscript:

ANOVA	Analysis of Variance
BVS	Bond Valence Sum
BSA	Bovine Serum Albumin
Chr	Chrysin
gCOSY	Gradient Correlation Spectroscopy
DMEM	Dulbecco’s modified Eagle’s medium
ESI-MS	Electron Spray Ionization Mass Spectrometry
FBS	Fetal Bovine Serum
FT-IR	Fourier Transform Infrared Spectroscopy
IC_50_	Half-maximal Inhibitory Concentration
gHSQC	Gradient Heteronuclear Single Quantum Coherence
MIC	Minimum Inhibitory Concentration
NMR	Nuclear Magnetic Resonance Spectroscopy
phen	1,10-Phenanthroline
PBS	Phosphate Buffer Saline
PRESAT	Presaturation
SD	Standard deviation
SEM	Standard error mean
Et_3_N	Triethylamine
UV–Visible	Ultraviolet–Visible Spectroscopy
XTT	2,3-bis-(2-methoxy-4-nitro-5-sulfophenyl)-2H-tetrazolium-5-carboxanilide
ZOI	Zone Of Inhibition

## References

[1] Sherwood E.R., Toliver-Kinsky T. (2004). Mechanisms of the inflammatory response. Best practice & research. Clin. Anaesthesiol..

[2] Nathan C., Ding A. (2010). Nonresolving inflammation. Cell.

[3] Chen L., Deng H., Cui H., Fang J., Zuo Z., Deng J., Li Y., Wang X., Zhao L. (2017). Inflammatory responses and inflammation-associated diseases in organs. Oncotarget.

[4] Sugimoto M.A., Sousa L.P., Pinho V., Perretti M., Teixeira M.M. (2016). Resolution of Inflammation: What Controls Its Onset?. Front. Immunol..

[5] Zhang W., Ruan J., Cheng J., Wang Y., Zheng Y., Lin M., Zhang Y., Wang T. (2025). In vitro anti-inflammatory terpenoid glycosides from the seeds of dolichos lablab. Molecules.

[6] Zappavigna S., Cossu A.M., Grimaldi A., Bocchetti M., Ferraro G.A., Nicoletti G.F., Filosa R., Caraglia M. (2020). Anti-inflammatory drugs as anticancer agents. Int. J. Mol. Sci..

[7] Tai F.W.D., McAlindon M.E. (2021). Non-steroidal anti-inflammatory drugs and the gastrointestinal tract. Clin. Med..

[8] Harirforoosh S., Asghar W., Jamali F. (2013). Adverse effects of nonsteroidal antiinflammatory drugs: An update of gastrointestinal, cardiovascular and renal complications. J. Pharm. Pharm. Sci..

[9] Bindu S., Mazumder S., Bandyopadhyay U. (2020). Non-steroidal anti-inflammatory drugs (NSAIDs) and organ damage: A current perspective. Biochem. Pharmacol..

[10] Goldkind L., Laine L. (2006). A systematic review of NSAIDs withdrawn from the market due to hepatotoxicity: Lessons learned from the bromfenac experience. Pharmacoepidemiol. Drug Saf..

[11] Acharya Y., Taneja K.K., Haldar J. (2023). Dual functional therapeutics: Mitigating bacterial infection and associated inflammation. RSC Med. Chem..

[12] Hu L., Luo Y., Yang J., Cheng C. (2025). Botanical Flavonoids: Efficacy, absorption, metabolism and advanced pharmaceutical technology for improving bioavailability. Molecules.

[13] Ullah A., Munir S., Badshah S.L., Khan N., Ghani L., Poulson B.G., Emwas A.-H., Jaremko M. (2020). Important flavonoids and their role as a therapeutic agent. Molecules.

[14] Panche A.N., Diwan A.D., Chandra S.R. (2016). Flavonoids: An Overview. J. Nutr. Sci..

[15] Naz S., Imran M., Rauf A., Orhan I.E., Shariati M.A., Iahtisham-Ul-Haq, IqraYasmin, Shahbaz M., Qaisrani T.B., Shah Z.A. (2019). Chrysin: Pharmacological and therapeutic properties. Life Sci..

[16] Sokal A., Mruczek P., Niedoba M., Dewalska A., Stocerz K., Kadela-Tomanek M. (2025). Anticancer activity of ether derivatives of chrysin. Molecules.

[17] Mani R., Natesan V. (2018). Chrysin: Sources, beneficial pharmacological activities, and molecular mechanism of action. Phytochemistry.

[18] Zhao J., Yang J., Xie Y. (2019). Improvement strategies for the oral bioavailability of poorly water-soluble flavonoids: An overview. Int. J. Pharm..

[19] Loginova N.V., Harbatsevich H.I., Osipovich N.P., Ksendzova G.A., Koval’chuk T.V., Polozov G.I. (2020). Metal complexes as promising agents for biomedical applications. Current Med. Chem..

[20] Yasir K.H., Parveen S., Yousuf I., Tabassum S., Arjmand F. (2022). Metal complexes of NSAIDs as potent anti-tumor chemotherapeutics: Mechanistic insights into cytotoxic activity via multiple pathways primarily by inhibition of COX–1 and COX–2 enzymes. Coord. Chem. Rev..

[21] Halevas E., Matsia S., Hatzidimitriou A., Geromichalou E., Papadopoulos T.A., Katsipis G., Pantazaki A., Litsardakis G., Salifoglou A. (2022). A unique ternary Ce(III)-quercetin-phenanthroline assembly with antioxidant and anti-inflammatory properties. J. Inorg. Biochem..

[22] Matsia S., Papadopoulos A., Hatzidimitriou A., Schumacher L., Koldemir A., Pöttgen R., Panagiotopoulou A., Chasapis C.T., Salifoglou A. (2025). Hybrid lanthanide metal–organic compounds with flavonoids: Magneto-optical properties and biological activity profiles. Int. J. Mol. Sci..

[23] Tsave O., Salifoglou A. (2021). Biomimetic activity of soluble, well-defined, aqueous Ti(IV)-citrate species toward adipogenesis. An in vitro study. J. Inorg. Biochem..

[24] Elie B.T., Fernández-Gallardo J., Curado N., Cornejo M.A., Ramos J.W., Contel M. (2019). Bimetallic titanocene-gold phosphane complexes inhibit invasion, metastasis, and angiogenesis-associated signaling molecules in renal cancer. Eur. J. Med. Chem..

[25] Etsè K.S., Harrad M.A., Etsè K.D., Zaragoza G., Demonceau A., Mouithys-Mickalad A. (2025). Free radical scavenging activity and inhibition of enzyme-catalyzed oxidation by trans-aryl-Palladium complexes. Molecules.

[26] McKinnon J.J., Spackman M.A., Mitchell A.S. (2004). Novel tools for visualizing and exploring intermolecular interactions in molecular crystals. Acta Crystallogr. B Struct. Sci..

[27] Ansari A.A. (2008). DFT and ^1^H NMR molecular spectroscopic studies on biologically anti-oxidant active paramagnetic lanthanide(III)-chrysin complexes. Main. Group. Chem..

[28] Matsia S., Lazopoulos G., Hatzidimitriou A., Reimann M.K., Pöttgen R., Salifoglou A. (2023). Chemical reactivity profile of rare earth metal ions with flavonoids. From structural speciation to magneto-optical properties. Polyhedron.

[29] Halevas E., Mavroidi B., Pelecanou M., Hatzidimitriou A. (2021). Structurally characterized zinc complexes of flavonoids chrysin and quercetin with antioxidant potential. Inorg. Chim. Acta.

[30] Ystenes M., Rytter E. (1992). Fourier transform infrared spectra of three titanium tetrachloride-ethyl benzoate complexes. Assignment based on five isotopic homologues and extension of the ethyl benzoate force field. Spectrochim. Acta Part. A Mol. Spectrosc..

[31] Kaushal R., Kumar N., Chaudhary A., Arora S., Awasthi P. (2014). Synthesis, spectral characterization, and antiproliferative studies of mixed ligand titanium complexes of adamantylamine. Bioinorg. Chem. Appl..

[32] Alem M.B., Desalegn T., Damena T., Bayle E.A., Koobotse M.O., Ngwira K.J., Ombito J.O., Zachariah M., Demissie T.B. (2023). Organic-inorganic hybrid salt and mixed ligand Cr(III) complexes containing the natural flavonoid chrysin: Synthesis, characterization, computational, and biological studies. Front. Chem..

[33] Halevas E., Mavroidi B., Antonoglou O., Hatzidimitriou A., Sagnou M., Pantazaki N., Litsardakis G., Pelecanou M. (2020). Structurally characterized gallium-chrysin complexes with anticancer potential. Dalton Trans..

[34] Khitrov G.A., Strouse G.F., Gaumet J.-J. (2004). Characterization of Ti_6_O_4_(O_2_C_4_H_5_)_8_(OCH_2_CH_3_)_8_ by electrospray time of flight mass spectrometry. J. Am. Soc. Mass Spectrom..

[35] Hwang S.H., Kim H.Y., Zuo G., Wang Z., Lee J.-Y., Lim S.S. (2018). Anti-glycation, carbonyl trapping and anti-inflammatory activities of chrysin derivatives. Molecules.

[36] Oggero J., Gasser F.B., Zacarías S.M., Burns P., Baravalle M.E., Renna M.S., Ortega H.H., Vaillard S.E., Vaillard V.A. (2023). PEGylation of Chrysin improves its water solubility while preserving the in vitro biological activity. J. Agric. Food Chem..

[37] Shrestha A., Pandey R.P., Dhakal D., Parajuli P., Sohnget J.K. (2018). Biosynthesis of flavone C-glucosides in engineered Escherichia coli. Appl. Microbiol. Biotechnol..

[38] Pusz J. (2001). The Titanium(IV), Iron(III) and Manganese(II) complexes of Chrysin-4′-sulfonate. Pol. J. Chem..

[39] Dimitrov G.D., Atanassova M.S. (2003). Synthesis and spectroscopic characterization of a complex of 1,10-phenanthroline with magnesium. Z. Anorg. Allg. Chem..

[40] Pazderski L., Pawlak T., Sitkowski J., Kozerski L., Szłyk E. (2010). ^1^H NMR assignment corrections and ^1^H, ^13^C, ^15^N NMR coordination shifts structural correlations in Fe(II), Ru(II) and Os(II) cationic complexes with 2,2′-bipyridine and 1,10-phenanthroline. Magn. Reson. Chem..

[41] Meléndez E. (2002). Titanium complexes in cancer treatment. Crit. Rev. Oncol. Hematol..

[42] Petanidis S., Kioseoglou E., Hadzopoulou-Cladaras M., Salifoglou A. (2013). Novel ternary vanadium-betaine-peroxido species suppresses H-ras and matrix metalloproteinase-2 expression by increasing reactive oxygen species-mediated apoptosis in cancer cells. Cancer Lett..

[43] Halevas E., Tsave O., Yavropoulou M.P., Hatzidimitriou A., Yovos J.G., Psycharis V., Gabriel C., Salifoglou A. (2015). Design, synthesis and characterization of novel binary V(V)-Schiff base materials linked with insulin-mimetic vanadium-induced differentiation of 3T3-L1 fibroblasts to adipocytes. Structure–function correlations at the molecular level. J. Inorg. Biochem..

[44] Gabriel C., Venetis J., Kaliva M., Raptopoulou C.P., Terzis A., Drouza C., Meier B., Voyiatzis G., Potamitis C., Salifoglou A. (2009). Probing for missing links in the binary and ternary V(V)–citrate–(H_2_O_2_) systems: Synthetic efforts and in vitro insulin mimetic activity studies. J. Inorg. Biochem..

[45] Tsave O., Yavropoulou M.P., Kafantari M., Gabriel C., Yovos J.G., Salifoglou A. (2018). Comparative assessment of metal-specific adipogenic activity in zinc and vanadium-citrates through associated gene expression. J. Inorg. Biochem..

[46] Salari N., Faraji F., Jafarpour S., Faraji F., Rasoulpoor S., Dokaneheifard S., Mohammadi M. (2022). Anti-cancer activity of Chrysin in cancer therapy: A systematic review. Indian. J. Surg. Oncol..

[47] Fu B., Xue J., Li Z., Shi X., Jiang B.-H., Fang J. (2007). Chrysin inhibits expression of hypoxia-inducible factor-1α through reducing hypoxia-inducible factor-1α stability and inhibiting its protein synthesis. Mol. Cancer Ther..

[48] Zhu Y., Yao X., Long J., Li R., Liu Y., Yang Z., Zheng X. (2019). Fluorine-containing chrysin derivatives: Synthesis and biological activity. Nat. Prod. Commun..

[49] Moghadam E.R., Ang H.L., Asnaf S.E., Zabolian A., Saleki H., Yavari M., Esmaeili H., Zarrabi A., Ashrafizadeh M., Kumar A.P. (2020). Broad-spectrum preclinical antitumor activity of chrysin: Current trends and future perspectives. Biomolecules.

[50] Stone T.W., Darlington L.G. (2017). Microbial carcinogenic toxins and dietary anti-cancer protectants. Cell. Mol. Life Sci..

[51] Katanov C., Lerrer S., Liubomirski Y., Leider-Trejo L., Meshel T., Jair B., Feniger-Barish R., Kamer I., Soria-Artzi G., Hadar K. (2015). Regulation of the inflammatory profile of stromal cells in human breast cancer: Prominent roles for TNF-α and the NF-κB pathway. Stem Cell Res. Ther..

[52] Fukata M., Michelsen K.S., Eri R., Thomas L.S., Hu B., Lukasek K., Nast C.C., Lechago J., Xu R., Naiki Y. (2005). Toll-like receptor-4 is required for intestinal response to epithelial injury and limiting bacterial translocation in a murine model of acute colitis. Am. J. Physiol. Gastrointest. Liver Physiol..

[53] Guo M., Guo Z., Sadler P.J. (2001). Titanium(IV) targets phosphoesters on nucleotides: Implications for the mechanism of action of the anticancer drug titanocene dichloride. J. Biol. Inorg. Chem..

[54] Erxleben A., Claffey J., Tacke M. (2010). Binding and hydrolysis studies of antitumoural titanocene dichloride and Titanocene Y with phosphate diesters. J. Inorg. Biochem..

[55] Wang H., Zhong J., Xiao K., Tian Z. (2018). Enrichment of intact phosphoproteins using immobilized titanium(IV) affinity chromatography microspheres. Sep. Sci. Plus.

[56] Christodoulou C.V., Eliopoulos A.G., Young L.S., Hodgkins L., Ferry D.R., Kerr D.J. (1998). Anti-proliferative activity and mechanism of action of titanocene dichloride. Br. J. Cancer.

[57] Olszewski U., Deally A., Tacke M., Hamilton G. (2012). Alterations of phosphoproteins in NCI-H526 small cell lung cancer cells involved in cytotoxicity of cisplatin and titanocene Y. Neoplasia.

[58] Brown I.D., Altermatt D. (1985). Bond-valence parameters obtained from a systematic analysis of the inorganic crystal structure database. Acta Cryst..

[59] Turner M.J., McKinnon J.J., Wolff S.K., Grimwood D.J., Spackman P.R., Jayatilaka D., Spackman M.A. (2017). Crystal Explorer 17.5.

[60] Spackman M.A., Jayatilaka D. (2009). Hirshfeld surface analysis. Cryst. Eng. Comm..

[61] Hirshfeld F.L. (1977). Bonded-Atom fragments for describing molecular charge densities. Theor. Chim. Acta.

[62] Clausen H.F., Chevallier M.S., Spackman M.A., Iversen B.B. (2010). Three new co-crystals of hydroquinone: Crystal structures and Hirshfeld surface analysis of intermolecular interactions. New J. Chem..

[63] Spackman M.A., McKinnon J.J. (2002). Fingerprinting intermolecular interactions in molecular crystals. Cryst. Eng. Comm..

[64] Parkin A., Barr G., Dong W., Gilmore C.J., Jayatilaka D., McKinnon J.J., Spackman M.A., Wilson C.C. (2007). Comparing entire crystal structures: Structural genetic fingerprinting. Cryst. Eng. Comm..

[65] Rohl A.L., Moret M., Kaminsky W., Claborn K., McKinnon J.J., Kahr B. (2008). Hirshfeld surfaces identify inadequacies in computations of intermolecular interactions in crystals: Pentamorphic 1,8-dihydroxyanthraquinone. Cryst. Growth Des..

[66] Allen F.H., Kennard O., Watson D.G., Brammer L., Orpen A.G., Taylor R.J. (1987). Tables of bond lengths determined by X-Ray and neutron diffraction. Part 1. Bond lengths in organic compounds. J. Chem. Soc. Perkin Trans..

[67] Bruker Analytical X–Ray Systems, Inc. (2006). Apex2, Version 2 User Manual.

[68] Siemens Industrial Automation Inc. (1996). SADABS: Area–Detector Absorption Correction.

[69] Palatinus L., Chapuis G. (2007). SUPERFLIP—A computer program for the solution of crystal structures by charge flipping in arbitrary dimensions. J. Appl. Crystallogr..

[70] Betteridge P.W., Carruthers J.R., Cooper R.I., Prout K., Watkin D.J. (2003). CRYSTALS version 12: Software for guided crystal structure analysis. J. Appl. Crystallogr..

[71] Elmehrath S., Ahsan K., Munawar N., Alzamly A., Nguyen H.L., Greish Y. (2024). Antibacterial efficacy of copper-based metal–organic frameworks against Escherichia coli and Lactobacillus. RSC Adv..

[72] Munir M.T., Pailhories H., Eveillard M., Irle M., Aviat F., Dubreil L., Federighi M., Belloncle C. (2020). Testing the antimicrobial characteristics of wood materials: A Review of methods. Antibiotics.

[73] Smeriglio A., D’Angelo V., Cacciola A., Ingegneri M., Raimondo F.M., Trombetta D., Germanò M.P. (2022). New insights on phytochemical features and biological properties of *Alnus glutinosa* Stem Bark. Plants.

[74] Denaro M., Smeriglio A., Trombetta D. (2021). Antioxidant and anti-inflammatory activity of citrus flavanones mix and its stability after in vitro simulated digestion. Antioxidants.

[75] Danna C., Bazzicalupo M., Ingegneri M., Smeriglio A., Trombetta D., Burlando B., Cornara L. (2022). Anti-inflammatory and wound healing properties of leaf and rhizome extracts from the medicinal plant *Peucedanum ostruthium* (L.) W. D. J. Koch. Molecules.

[76] Cornara L., Ambu G., Alberto A., Trombetta D., Smeriglio A. (2022). Characterization of ingredients incorporated in the traditional mixed-salad of the capuchin monks. Plants.

[77] Smeriglio A., Denaro M., D’Angelo V., Germanò M.P., Trombetta D. (2020). Antioxidant, anti-inflammatory and anti-angiogenic properties of citrus lumia juice. Front. Pharmacol..

[78] Lazopoulos G., Matsia S., Maroulis M., Salifoglou A. (2025). *Cornus mas* L. extracts exhibit neuroprotective properties, further enhanced by metal-bound energy-linked organic substrates. Int. J. Mol. Sci..

