# Supplementary material for: Structural Speciation of Hybrid Ti(IV)-Chrysin Systems—Biological Profiling and Antibacterial, Anti-Inflammatory, and Tissue-Specific Anticancer Activity
Source: Molecules. 2025 Sep 9;30(18):3667. doi: 10.3390/molecules30183667 (PMC12472876; doi:10.3390/molecules30183667)
Supplement: Supplementary file 1 [file molecules-30-03667-s001.zip › molecules-3799185-supplementary.pdf]

Supplementary Information

Structural speciation of hybrid Ti(IV)-chrysin systems. Biological profiling, antibacterial, anti-inflammatory, and tissue-specific anticancer activity.

Sevasti Matsia [1], Georgios Lazopoulos [1], Antonios Hatzidimitriou [2], and Athanasios Salifoglou [1,*]

1 Laboratory of Inorganic Chemistry and Advanced Materials, School of Chemical Engineering, Aristotle University of Thessaloniki, Thessaloniki 54124, Greece
2 Laboratory of Inorganic Chemistry, School of Chemistry, Aristotle University of Thessaloniki, Thessaloniki 54124, Greece
* salif@auth.gr; Tel.:+30-2310-996-179

Table S1. Absolute error calculations based on m/z values of ESI-MS spectrometric measurements

Formula	Experimental values	Simulated values*	Absolute errors
M1_1 = [M1-Cl-CH_3OH-H]$^-$=[$C_{17}H_{15}TiO_6$]$^+$	363.0313	363.0343	0.0030
M1_2 = [M1+4CH_3O+Cl-5H]=[$C_{22}H_{27}TiCl_2O_{11}$]$^{9-}$	585.0615	585.0440	0.0175
M2_1 = [M2/4-phen-O+2CH_3O]=[$C_{17}H_{15}TiO_6$]$^+$	363.0323	363.0343	0.0020
M2_2 = [M2/4]=[$C_{27}H_{17}TiN_2O_5$]$^+$	497.0586	497.0611	0.0025
M2_3 = [M2/2-phen-H]=[$C_{42}H_{25}Ti_2N_2O_{10}$]$^+$	813.0449	813.0463	0.0014
M2_4 = [M2/2-H]=[$C_{54}H_{33}Ti_2N_4O_{10}$]$^+$	993.6147	993.1150	0.4997
M2_5 = [3M2/4-O-3phen+3CH_3OH-H]=[$C_{48}H_{38}Ti_3O_{17}$]$^{4+}$	1030.6084	1030.0542	0.5542
M2_6 = [M2-2phen-2Chr+7H]=[$C_{54}H_{41}Ti_4N_4O_{12}$]$^{13+}$	1129.0294	1129.0633	0.0339

* All simulated values have been calculated using the Xcalibur software

Table S2. ^{1}H-NMR shifts in DMSO-d^{6}, 600 MHz

Chrysin		Compound 1	Compound 2
Hydrogen type		δ shift (multiplicity)	
5-OH	12.85 (s)	---	---
7-OH	10.95 (broad band)	11.09 (s)	11.12 (s)
H(2′) and H(6′)	8.09 (d)	8.11 (d)	7.99 (m)
H(3′), H(4′), H(5′)	7.59 (m)	7.59 (m)	7.59 (m)
H(3)	7.01 (s)	7.01 (s)	7.00 (s)
H(8)	6.55 (s)	6.59 (s)	6.58 (s)
H(6)	6.24 (s)	6.27 (s)	6.27 (s)
CH_3OH	---	3.19 (s)	---
DMSO	2.53 (s)	2.53 (s)	2.54 (s)
**phen**		δ shift (multiplicity)	
H(11) and H(20)	9.13 (m)	---	9.22 (m)
H(13) and H(18)	8.55 (m)	---	8.77 (m)
H(15) and H(16)	8.02 (s)	---	8.19 (s)
H(12) and H(19)	7.8 (m)	---	8.01 (m)

Table S3. ^{13}C-NMR shifts in DMSO-d^{6}, 600 MHz

Chrysin		Compound 1	Compound 2
Carbon type		δ shift (multiplicity)	
C(4)	182.30 (s)	183.71 (s)	183.59 (s)
C(7)	165.01 (s)	166.33 (s)	168.49 (s)
C(2)	163.59 (s)	164.99 (s)	166.49 (s)
C(5)	161.88 (s)	163.24 (s)	162.99 (s)
C(9)	157.84 (s)	159.28 (s)	158.84 (s)
C(4′)	132.48 (s)	133.87 (s)	133.52 (s)
C(1′)	131.14 (s)	132.51 (s)	130.84 (s)
C(3′) and C(5′)	129.61 (s)	130.99 (s)	128.47 (s)
C(2′) and C(6′)	126.85 (s)	128.24 (s)	125.79 (s)
C(3)	105.59 (s)	106.98 (s)	106.33 (s)
C(10)	104.35 (s)	105.74 (s)	104.62 (s)
C(6)	99.48 (s)	100.87 (s)	99.00 (s)
C(8)	94.58 (s)	95.98 (s)	96.07 (s)
CH_3O^-	---	81.00 (s)	---
CH_3OH	---	50.24 (s)	---
DMSO	39.50 (m)	40.94 (m)	40.95 (m)
**phen**		δ shift (multiplicity)	
C(11) and C(20)	151.54 (s)	---	150.86 (s)
C(21) and C(22)	145.04 (s)	---	143.21 (s)
C(13) and C(18)	141.16 (s)	---	140.52 (s)
C(12) and C(19)	130.33 (s)	---	130.98 (s)
C(14) and C(17)	126.64 (s)	---	128.80 (s)
C(15) and C(16)	121.86 (s)	---	127.90 (s)

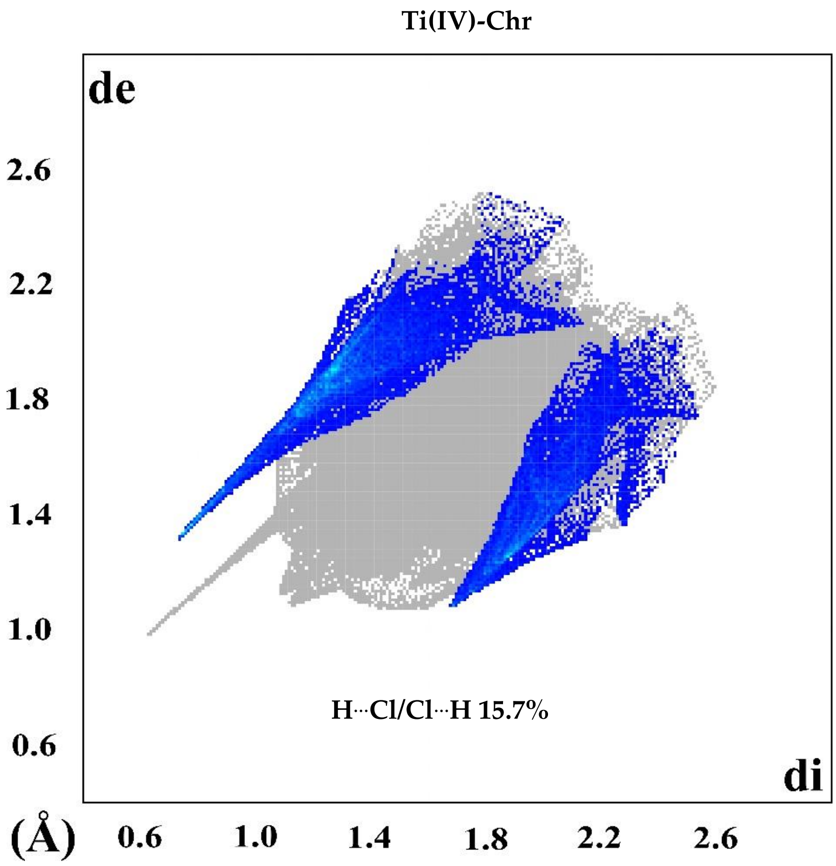

Figure S1. 2D Fingerprint of H···Cl/Cl···H interactions for **Ti(IV)-Chr** (**1**)

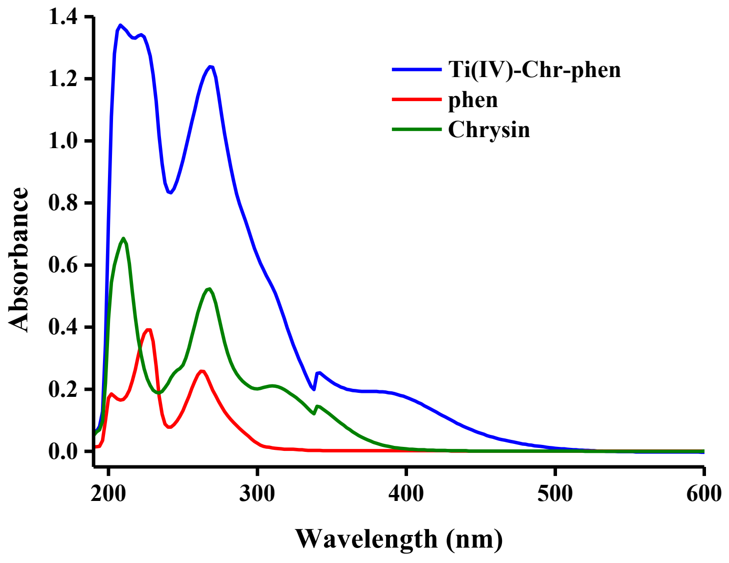

A

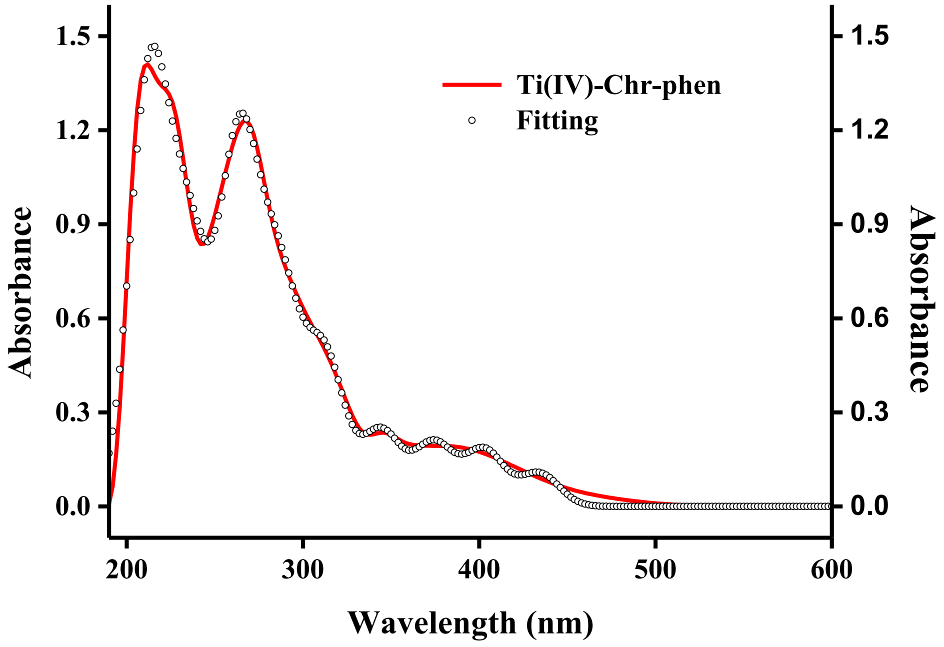

B

Figure S2. A. UV-Visible spectra of **2** compared to Chr and phen in methanol at 8×10^{-6} M; **B.** Fitting spectra of **2**.

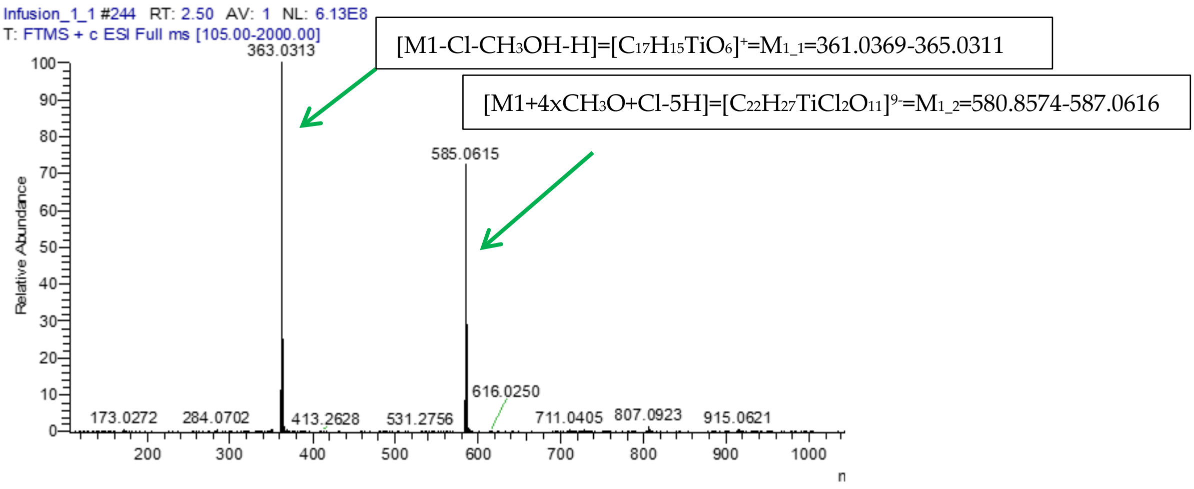

A

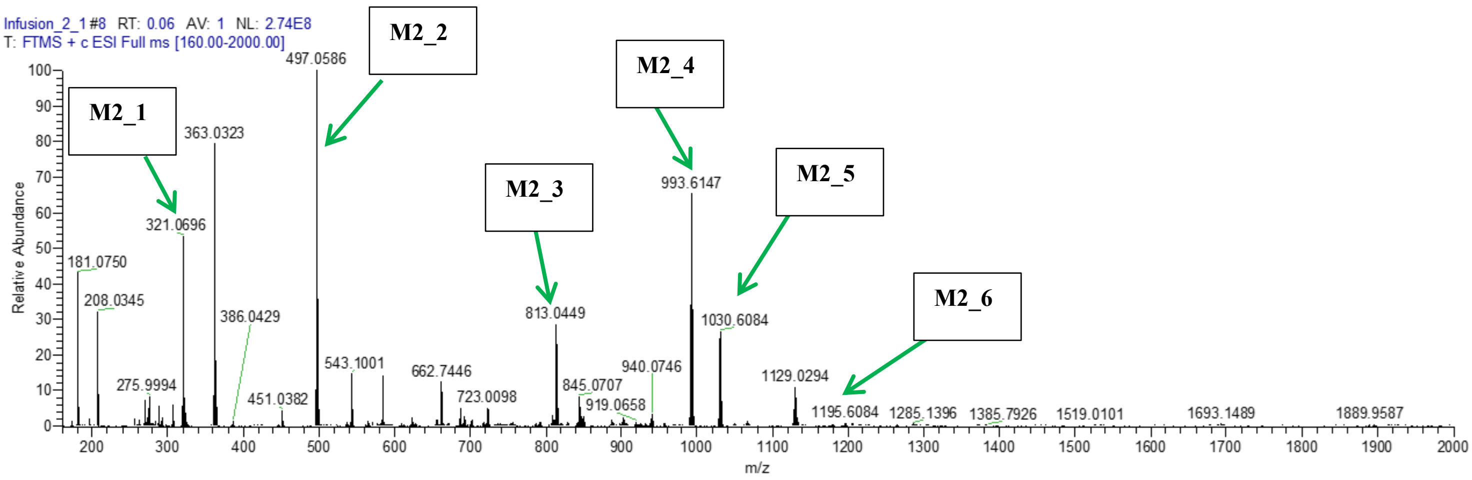

B

Figure S3. ESI-MS species detected in methanolic solution of **A)** **1; B) 2**

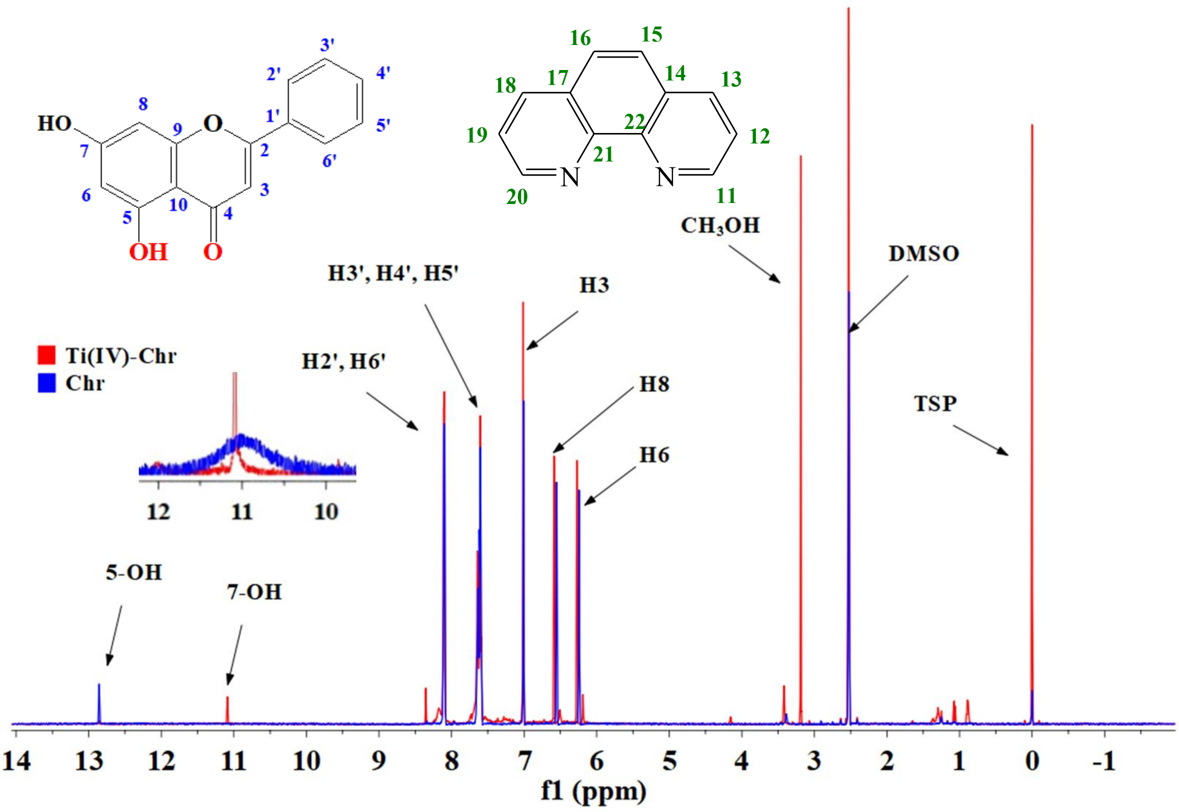

A

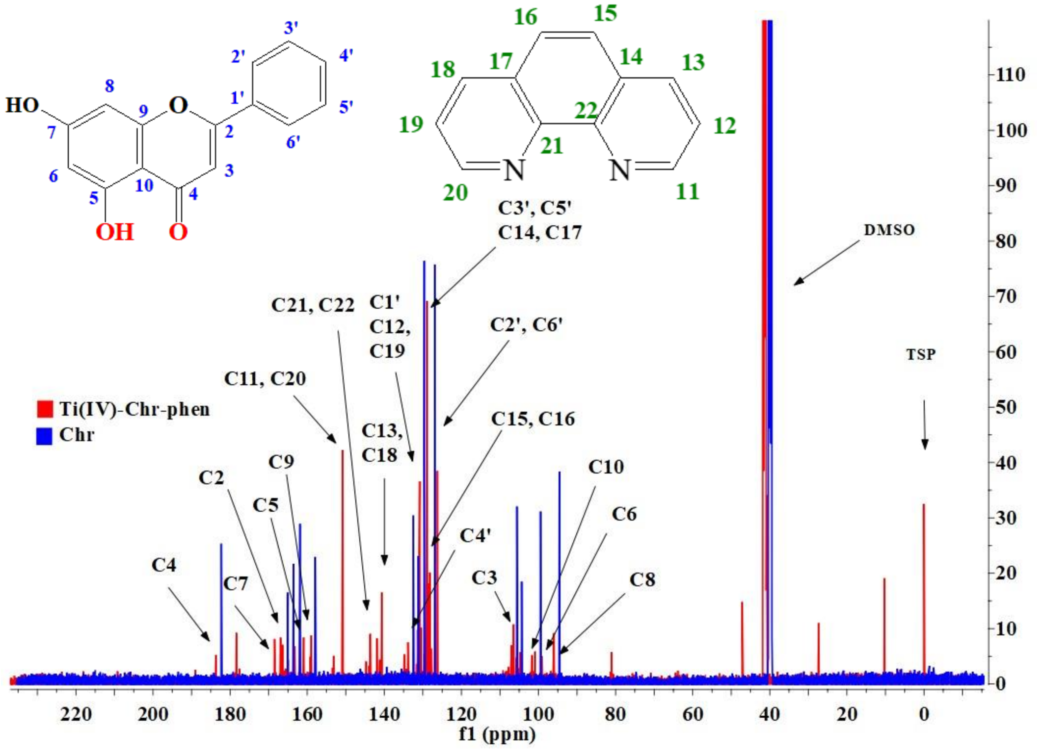

B

Figure S4. **A.** ^{1}H NMR spectra of **2**; **B.** ^{13}C NMR spectra of **2**; recorded in DMSO-d[6]

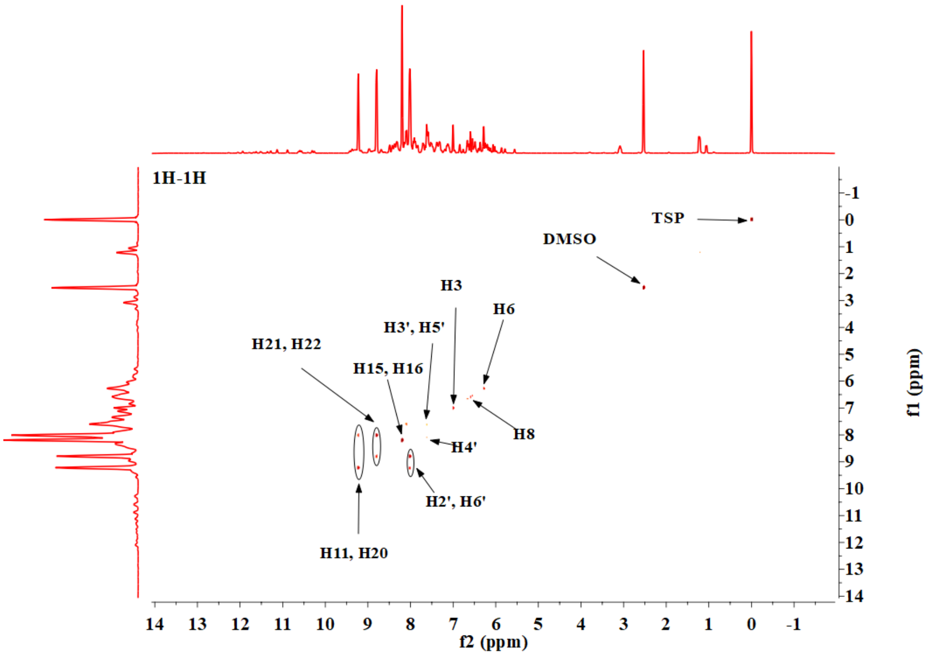

A

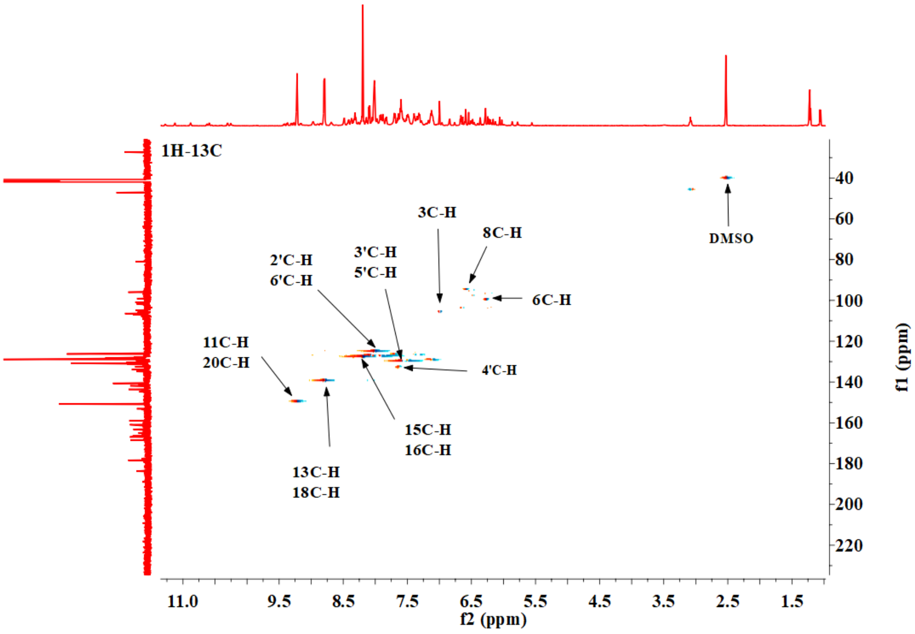

B

Figure S5. **A.** ^{1}H-^{1}H correlation through gCOSY NMR of **2**; **B.** ^{1}H-^{13}C correlation through gHSQC NMR of **2**

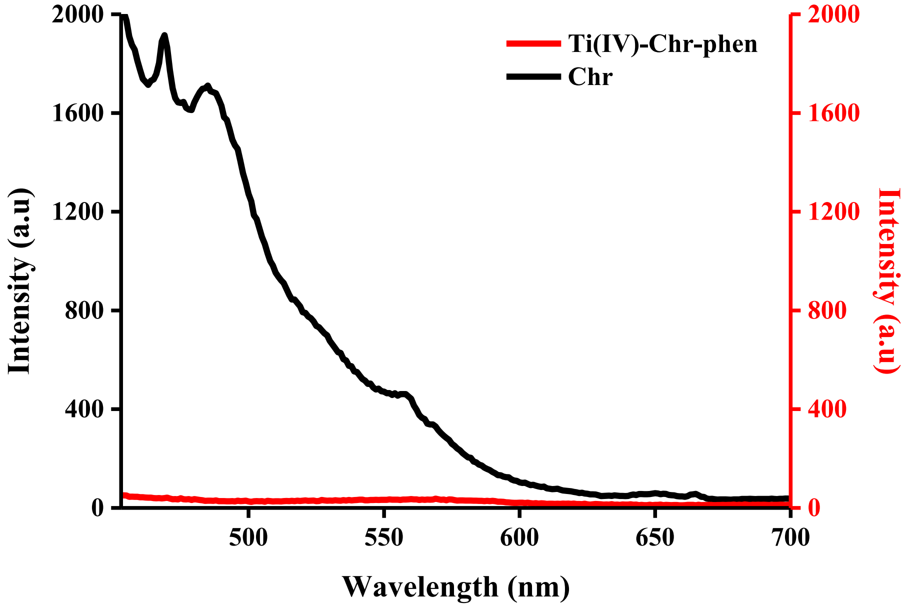

A

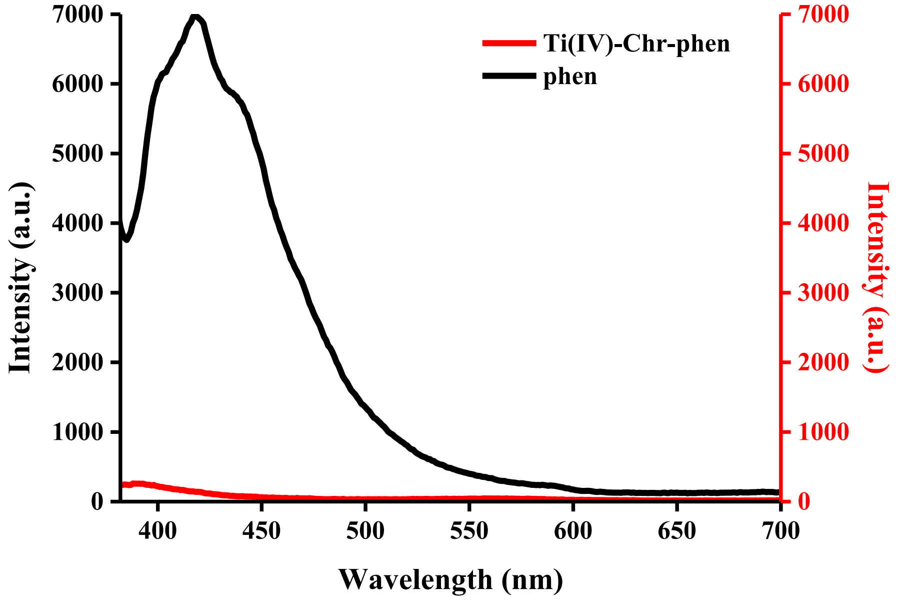

B

Figure S6. Solid-state normalized luminescence spectra of **2** compared to **A.** Chr; **B.** phen

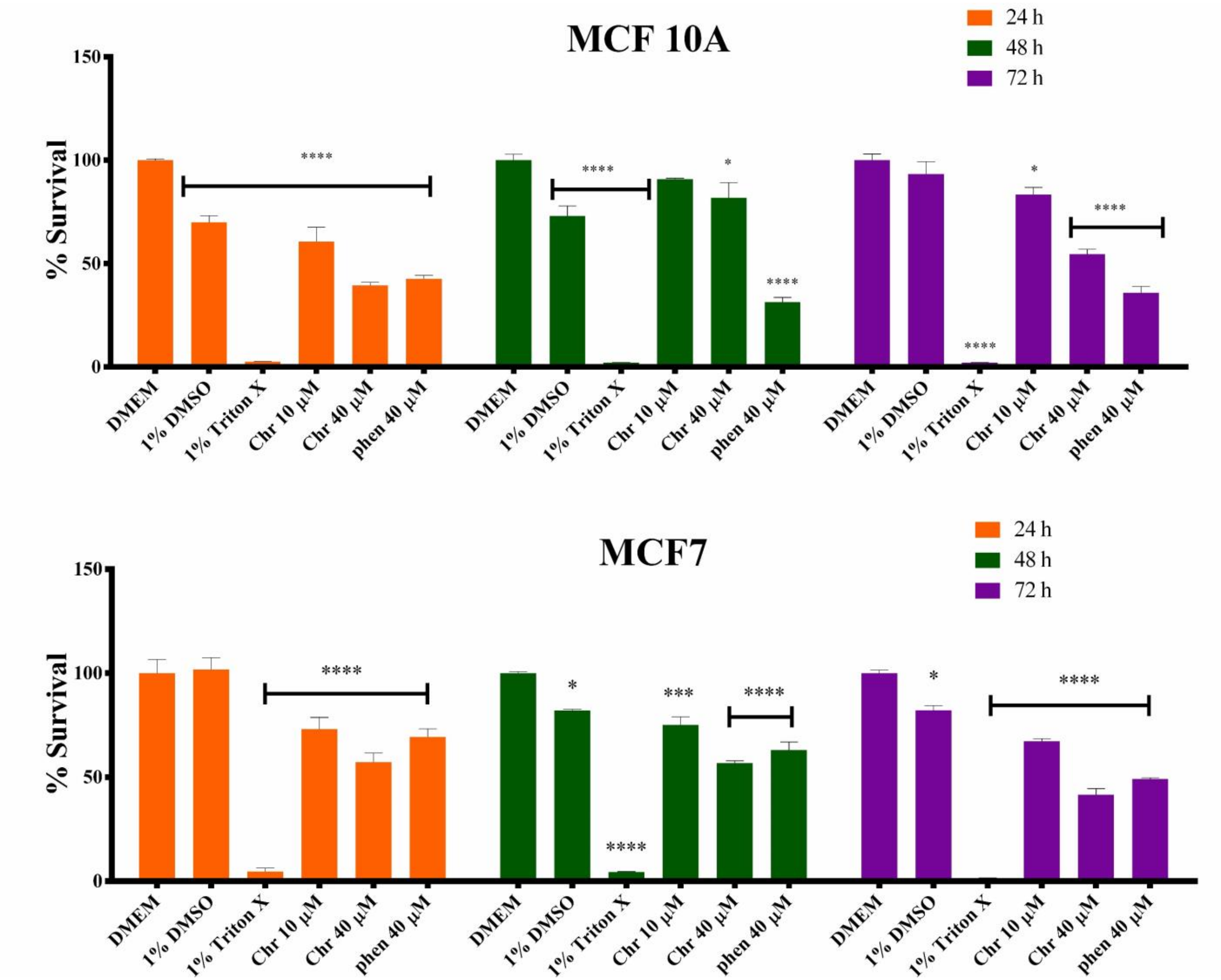

Figure S7. Viability assessment for Chr and phen, in physiological (MCF10A) and cancer (MCF7) human lung tissue cell cultures. Significance levels were assessed as follows: * $p < 0.05$ (significant), ** $p < 0.01$ (highly significant), *** $p < 0.001$ (extremely significant) and **** $p \leq 0.0001$ (extremely significant).

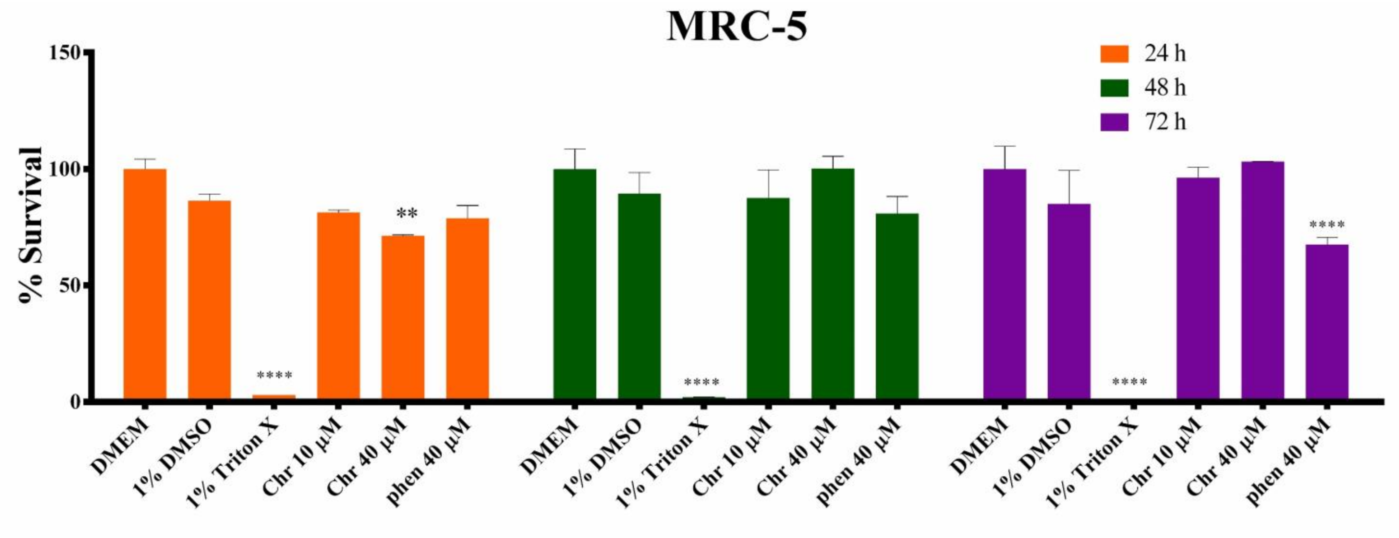

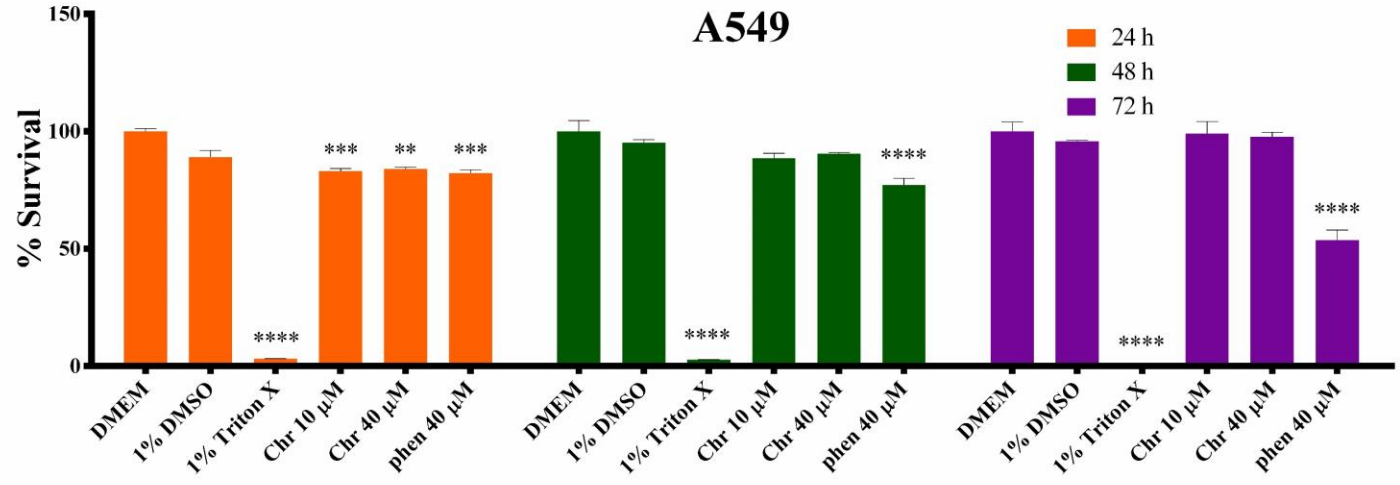

Figure S8. Viability assessment for Chr and phen, in physiological (MRC-5) and cancer (A549) human lung tissue cell cultures. Significance levels were assessed as follows: * $p < 0.05$ (significant), ** $p < 0.01$ (highly significant), *** $p < 0.001$ (extremely significant) and **** $p \leq 0.0001$ (extremely significant).

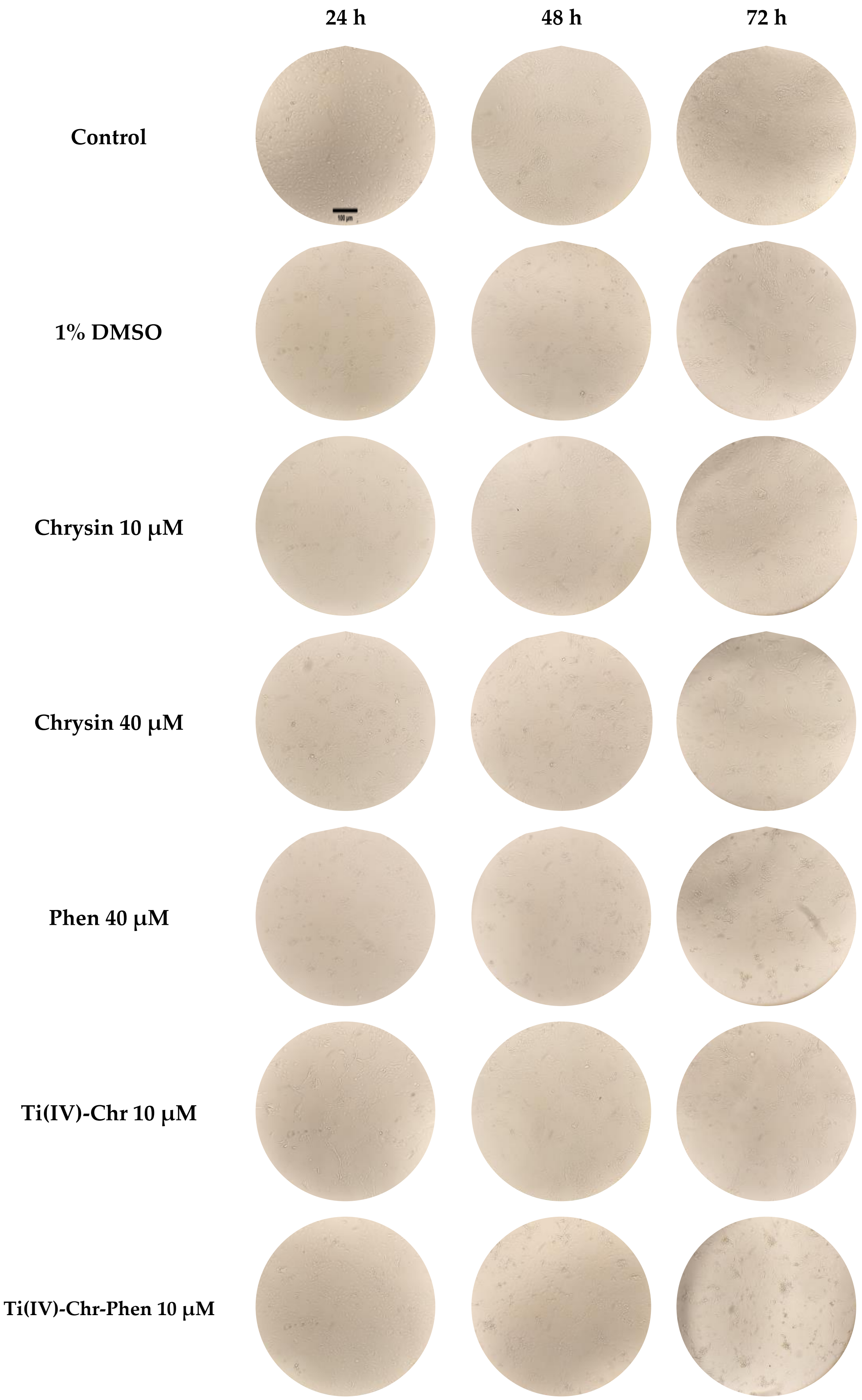

Figure S9. Cell morphology evaluation of MCF10A cultures after treatment with compounds **1** and **2**, and the corresponding ligands for 24, 48 and 72 h

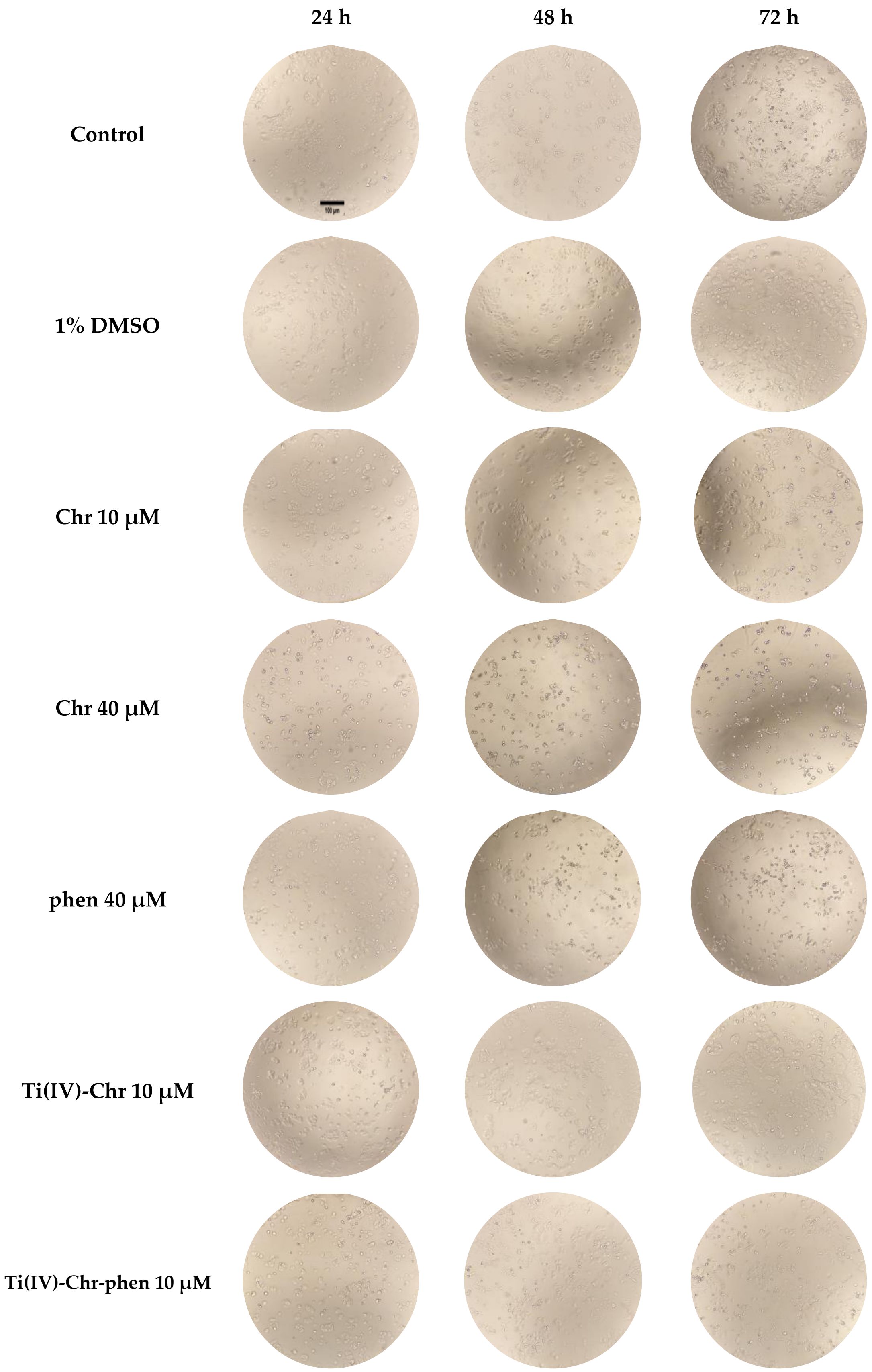

Figure S10. Cell Morphology evaluation of MCF7 cultures after treatment with compounds **1** and **2**, and the corresponding ligands for 24, 48 and 72 h

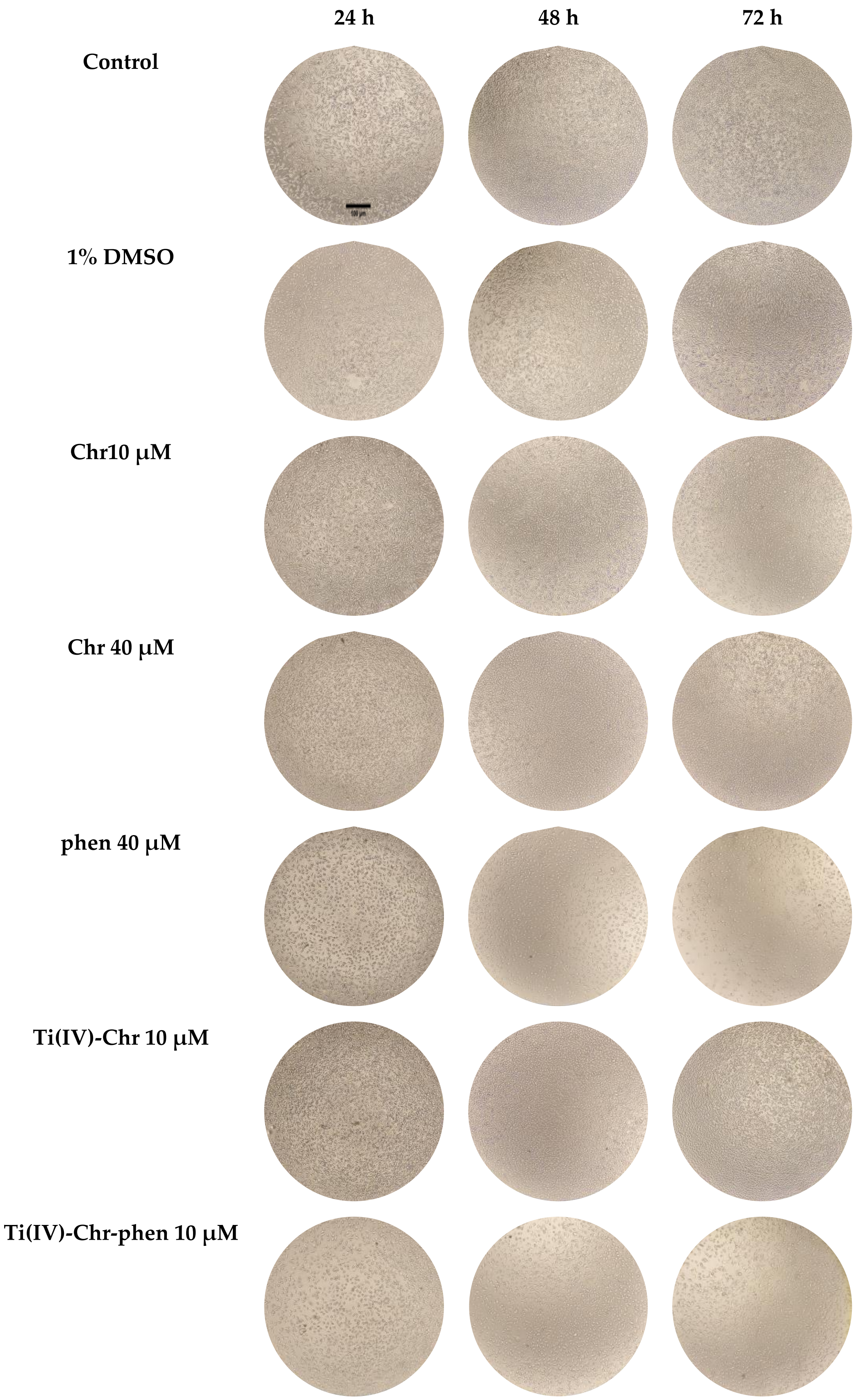

Figure S11. Cell morphological evaluation of MRC-5 cultures after treatment with compounds **1** and **2**, and the corresponding ligands for 24, 48 and 72 h

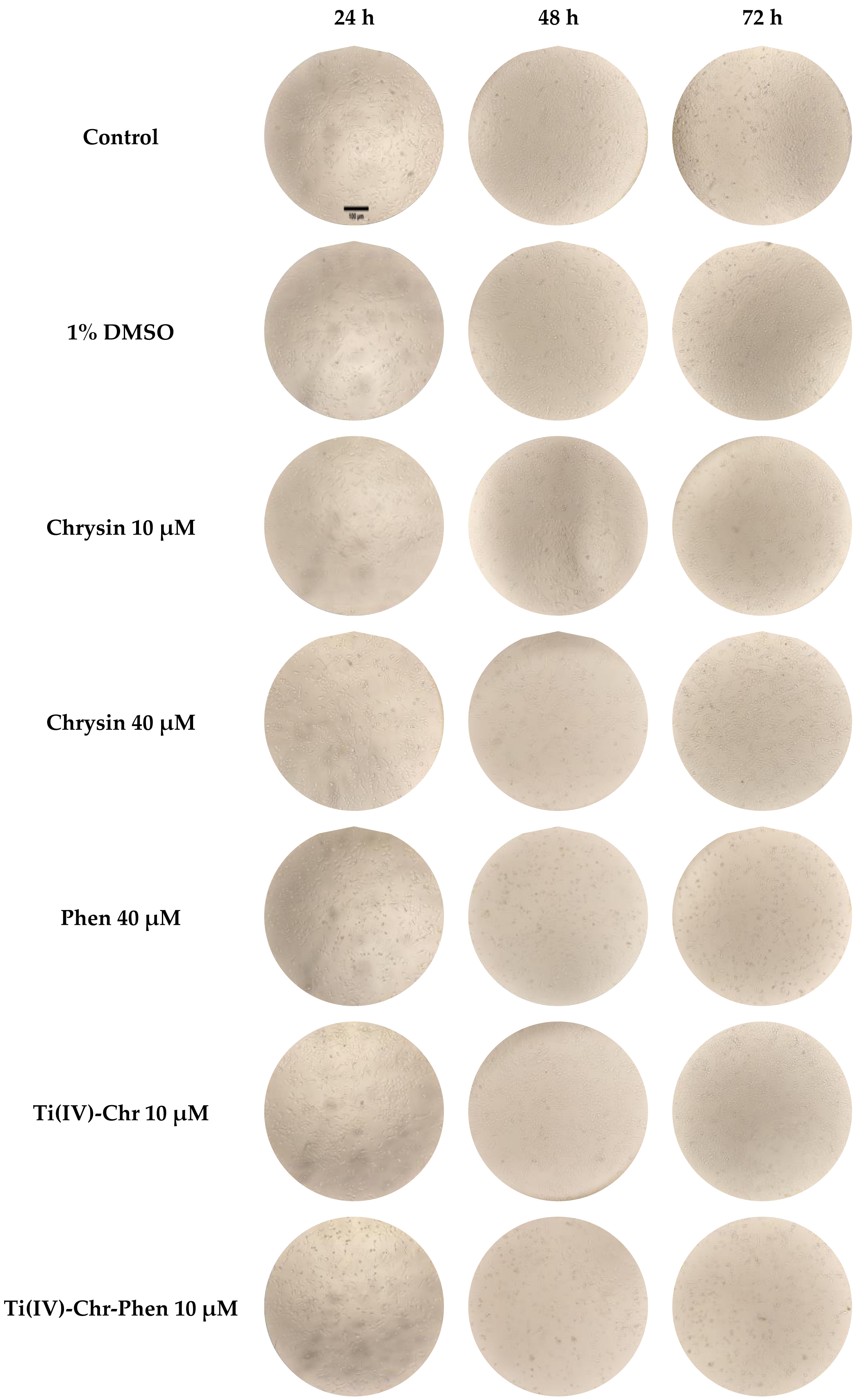

Figure S12. Cell morphology evaluation of A549 cultures after treatment with compounds **1** and **2**, and the corresponding ligands for 24, 48 and 72 h
